# Supplementary figures and images for: Dietary restriction shapes intergenerational ribosome abundance and early growth of Caenorhabditis elegans offspring
Source: PLoS Biol. 2026 Apr 9;24(4):e3003692. doi: 10.1371/journal.pbio.3003692 (PMC13065021; doi:10.1371/journal.pbio.3003692)

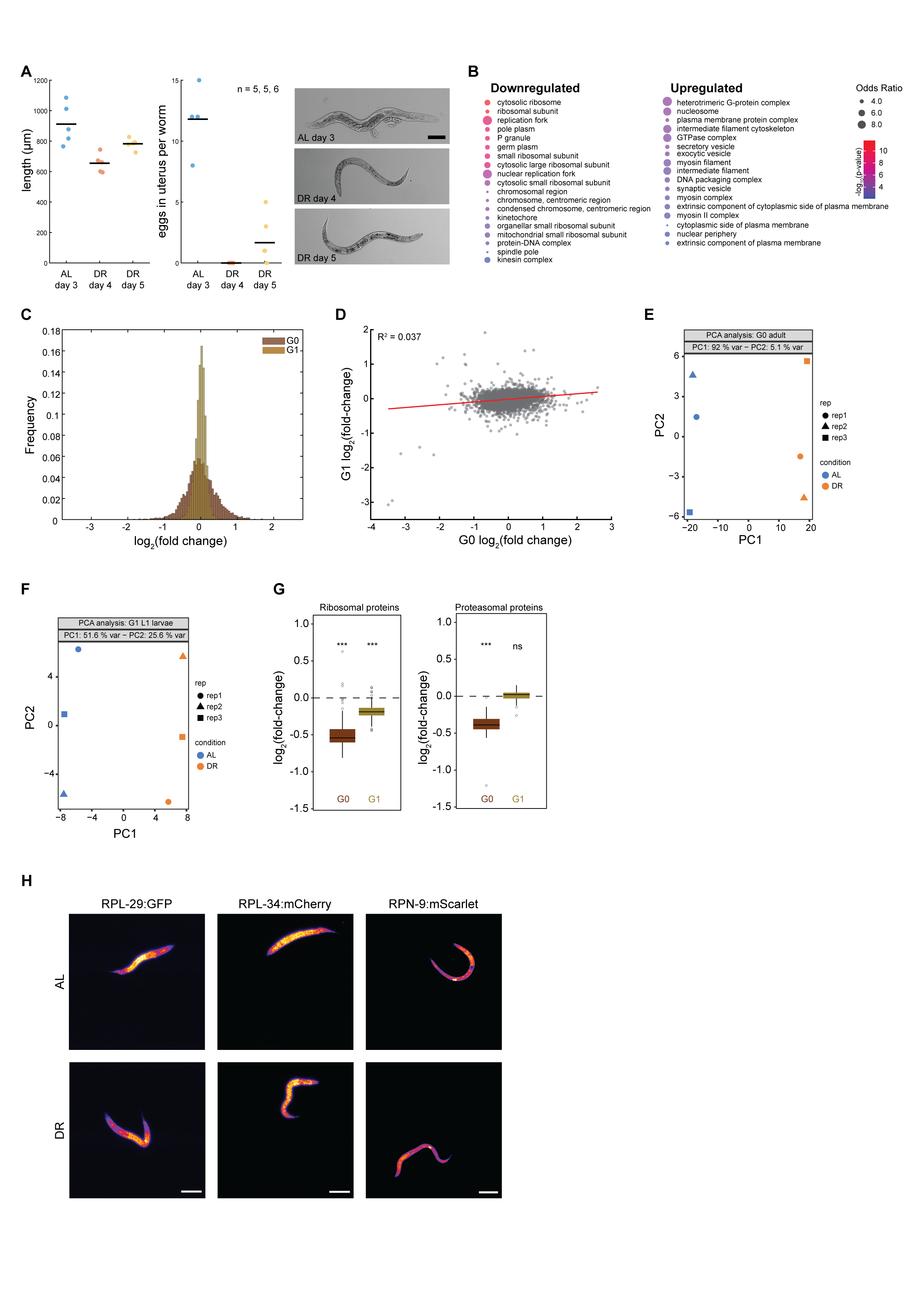

Supplement: S1 Fig — (A) Quantification of maternal growth under DR and AL. Animals were sampled on days 3, 4, and 5 after cultivation in liquid medium with 2*108 cfu/ml (DR) or 109 cfu/ml (AL). Length (left) and number of eggs inside uterus (right) were measured for ≥5 individuals per condition. DR significantly reduces size and number of embryos in uterus (length: p = 0.002 for AL day 3 vs. DR day 4; p = 0.02 for AL day 3 vs. DR day 5; number of eggs in uterus: p = 2.75*10−6 and 1.74*10−5, one-sided t test). No eggs were detected in DR animals after 4 days. Images show representative individuals at indicated sampling day. Scale bar = 100 μm. (B) Gene Ontology cellular component terms enriched among significantly downregulated (left) and upregulated (right) proteins in G0 adults under DR compared to AL conditions (FDR ≤ 0.05, minimum 2-fold change). Circle size indicates odds ratio and color intensity indicates significance, using an enrichment threshold of odds ratio > 3 and adjusted p-value < 0.01 (Benjamini-Hochberg correction). (C) Histograms comparing log2(fold change) in protein abundance between DR and AL conditions for G0 adults (blue) and G1 L1 larvae (orange). G0 adults show a broader distribution of fold changes (wider histogram) compared to G1 larvae, demonstrating larger proteome changes in the parental generation than in their progeny (mean absolute log2 fold change = 0.3 in G0 versus 0.11 in G1; variance = 0.078 in G0 versus 0.016 in G1). (D) Correlation between protein fold changes in G0 adults versus G1 L1 larvae (R2 = 0.037), demonstrating weak inheritance of proteome changes across generations. (E) Principal component analysis (PCA) of protein abundance data from G0 adults, showing separation between AL and DR conditions across 3 replicates. (F) PCA of protein abundance data from G1 L1 larvae showing separation between progeny of AL and DR parents across 3 replicates. (G) Box plots showing log2(fold change) in abundance of ribosomal proteins (left) and proteasomal pr [file pbio.3003692.s001.tif]

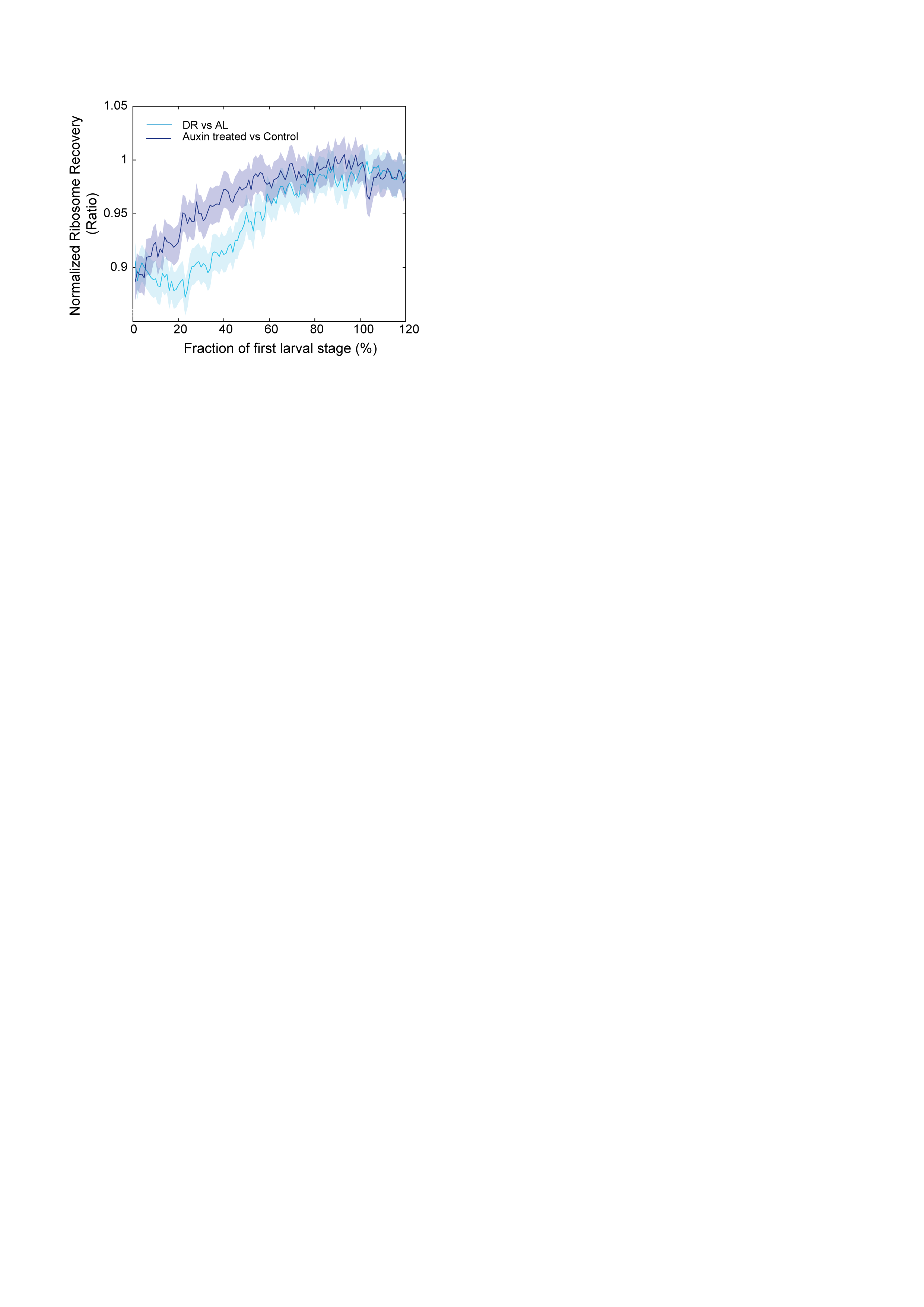

Supplement: S2 Fig — Ribosomal protein levels relative to control during L1 development after ribosomal protein depletion in the maternal proximal germline (dark blue), or after maternal DR (light blue). A value of 1 indicates full recovery of ribosomal protein levels compared to control animals. The recovery occurs faster after auxin-induced ribosomal protein depletion than after maternal DR. Solid lines: mean, shaded regions: 95% confidence interval. See S5 Data. (TIF) [file pbio.3003692.s002.tif]

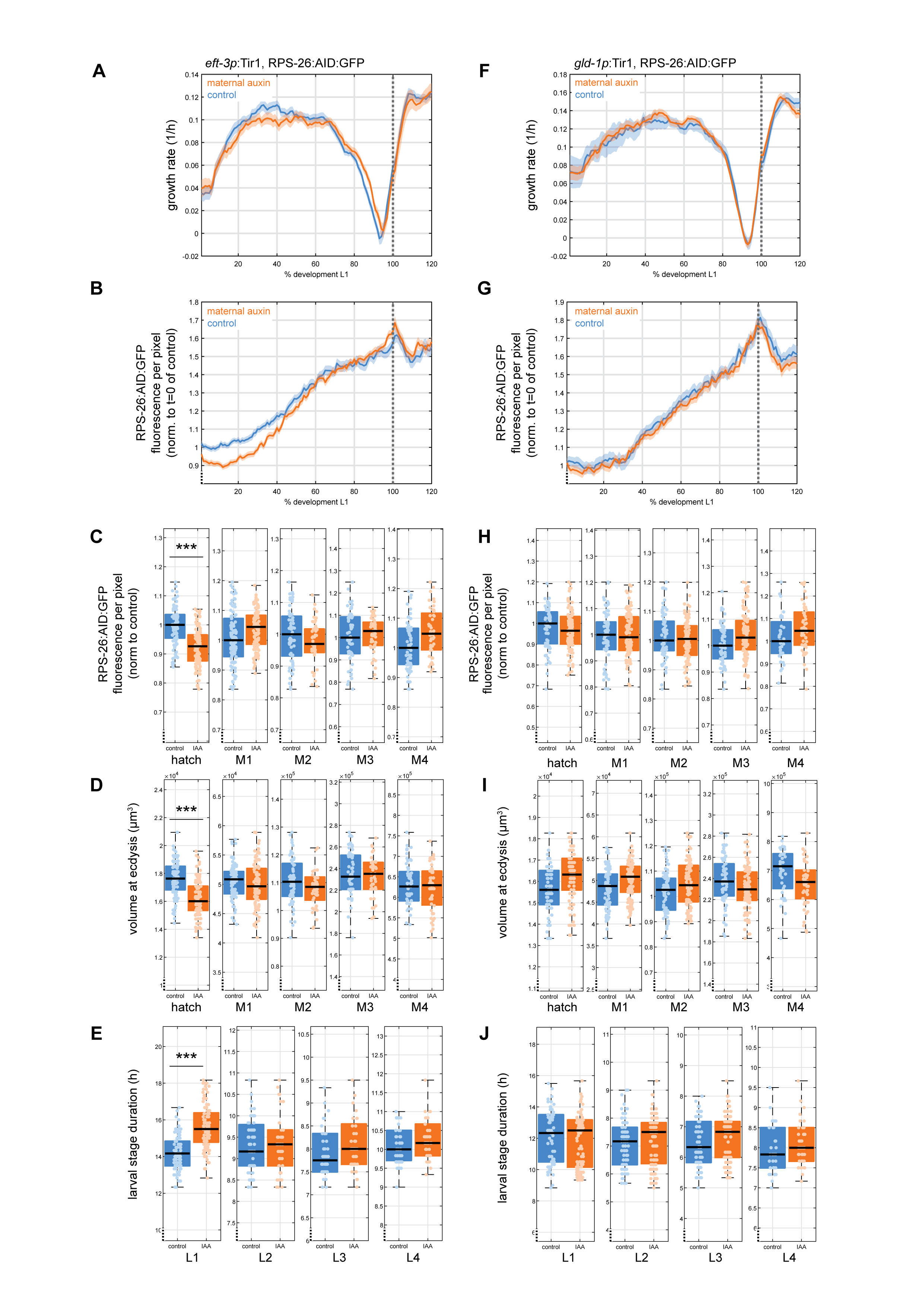

Supplement: S3 Fig — (A) Growth rate during L1 development for rps-26:aid:gfp, eft-3p:tir1 maternally auxin-treated progeny. (B) As (A), but RPS-26:AID:GFP fluorescence per pixel. (C–E) Fluorescence at hatch or ecdysis, volume at hatch or ecdysis, and larval stage duration. n ≥ 48 from 2 days (F–J) As (A–E), but for gld-1p:tir1. central line: median, box: interquartile range (IQR), whisker: ranges except extreme outliers (>1.5*IQR), individual values: crosses, extreme outliers: circles. Total number of individuals n ≥ 36 from 2 days, *** p < 10−10 (Wilcoxon rank-sum test). Precise p-values and sample size in S2 Table. See S5 Data. (TIF) [file pbio.3003692.s003.tif]

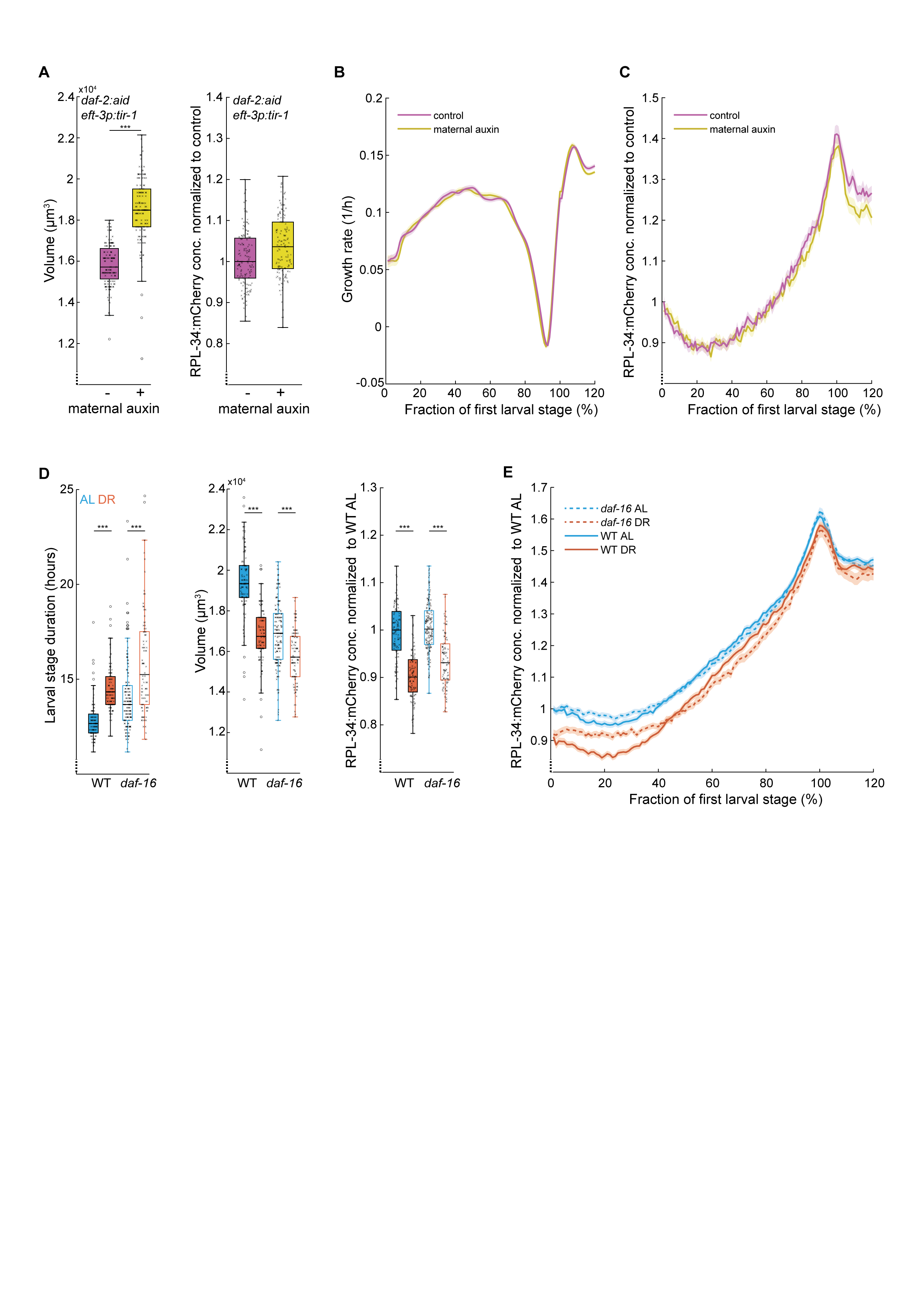

Supplement: S4 Fig — (A) Volume and RPL-34:mCherry concentration (pixel intensity normalized to control) at hatch of L1 progeny from mothers depleted for DAF-2:AID with 500 μM auxin from L4 stage onwards (yellow) and progeny of control animals (magenta). Progeny of DAF-2 depleted mothers are larger than progeny of control animals. This size increase is consistent with the increased size at hatch of daf-2(e1370) animals and after maternal somatic daf-2 RNAi(14), validating effective DAF-2 depletion by AID. Maternal auxin does not reduce progeny RPL-34:mCherry concentration but slightly increases it (p = 0.001, Wilcoxon rank-sum test). central line: median, box: interquartile range (IQR), whisker: ranges except extreme outliers (>1.5*IQR), individual values: crosses, extreme outliers: circles. Number of individuals n = 138 and 141 from days. p = 9*10-37 (Wilcoxon rank-sum test). (B) Growth rate of progeny during L1 development for parental depletion of DAF-2 and control. Individual trajectories were aligned at hatch point and M1 and re-scaled before averaging. Range between 100% and 120% represents the beginning of L2. Solid lines: mean, shaded regions: 95% confidence interval. Number of individuals and biological replicates as in (A). (C) As (B), but for RPL-34:mCherry concentration (fluorescence per pixel). (D) L1 larval stage duration, volume at hatching, and RPL-34:mCherry after maternal AL and DR in wild type (filled boxes) and daf-16(mu86) mutants (open boxes). daf-16(mu86) mutants are sensitive to maternal DR regarding their developmental rate and have altered volume at hatch, but these effects do not involve changes in the RPL-34:mCherry concentration. For each condition a total of at least n ≥ 100 individuals were measured on at least 3 days. *** indicate p < 10-5 (Wilcoxon rank-sum test). See S2 Table for precise sample size and p-values. (E) RPL-34:mCherry concentration as described for (C), but for indicated genotypes and maternal dietary treatments. Although daf-16(mu86) diff [file pbio.3003692.s004.tif]

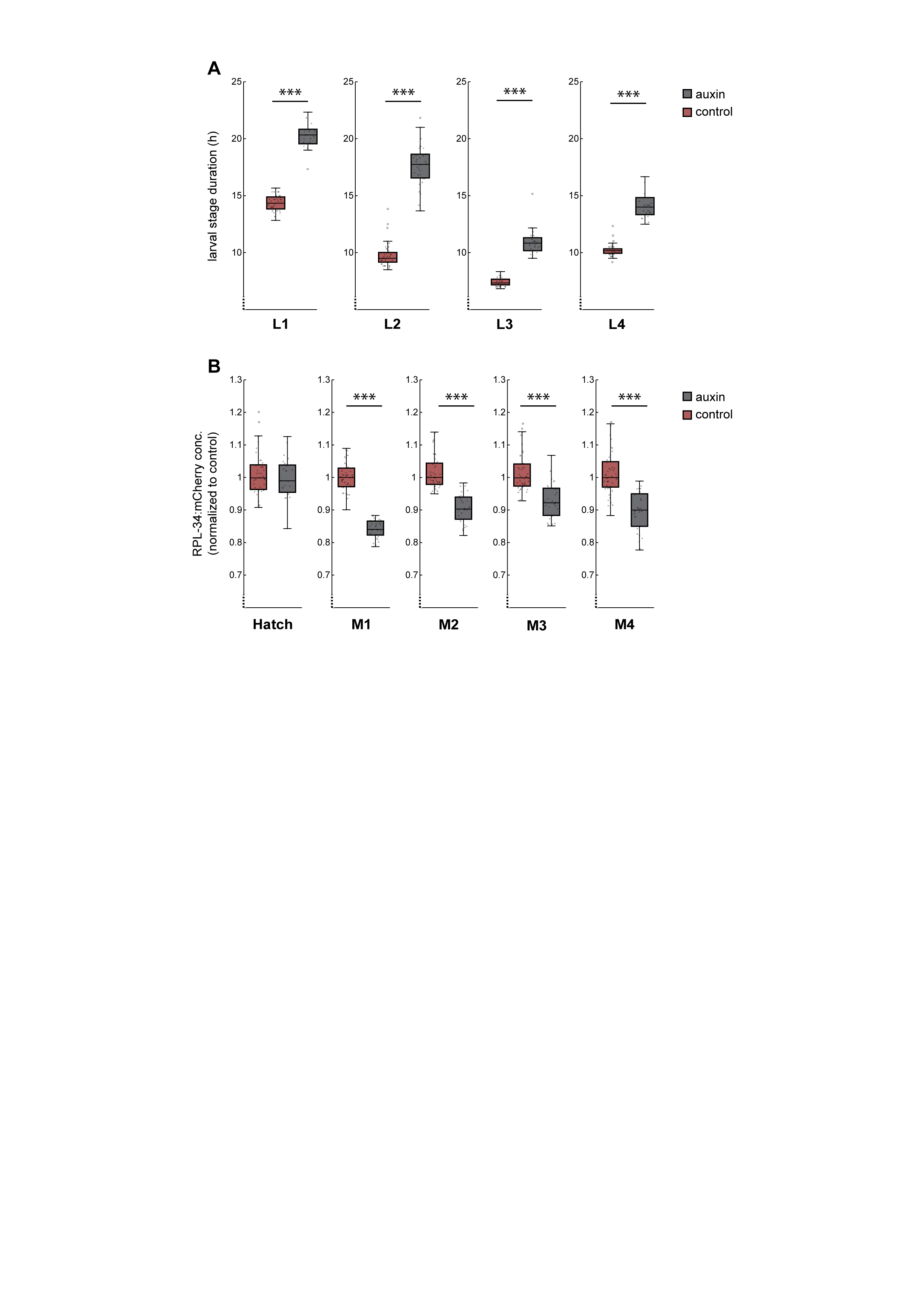

Supplement: S5 Fig — (A) Larval stage duration of raga-1:gfp:aid; eft-3p:tir-1; rpl-34:mCherry strain treated with 500 μM auxin (gray) and control (red). central line: median, box: interquartile ranges (IQR), whisker: ranges except extreme outliers (>1.5*IQR), individual values: crosses, extreme outliers: circles. Number of individuals n ≥ 41 from one day. (B) As (A), but for RPL-34:mCherry concentration (intensity per pixel normalized to control). *** indicate p < 10−5 (Wilcoxon rank sum test). See S2 Table for precise sample size and p-values. See S5 Data. (TIF) [file pbio.3003692.s005.tif]

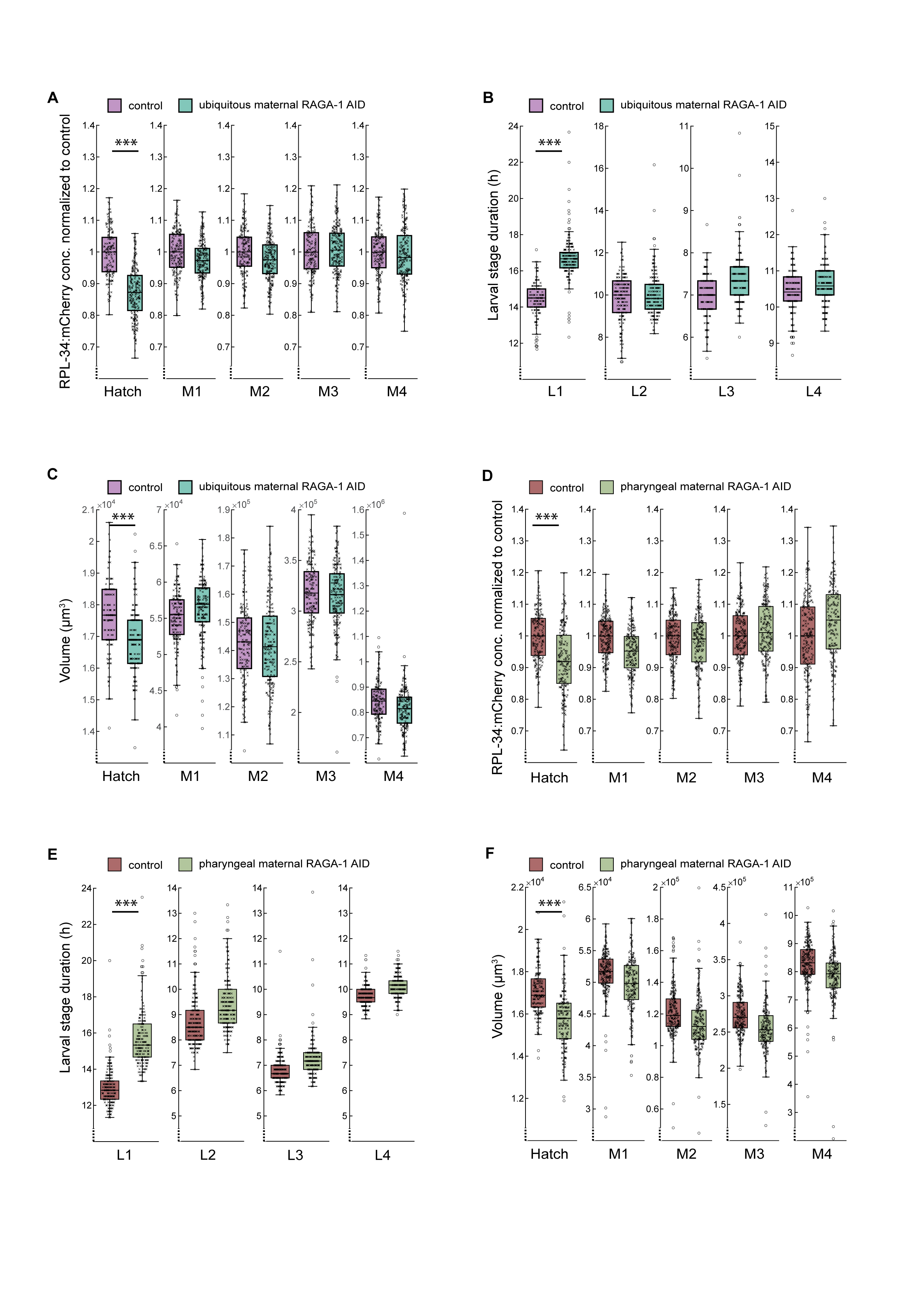

Supplement: S6 Fig — (A) RPL-34:mCherry concentration (intensity per pixel normalized to control) of raga-1:gfp:aid; eft-3p:tir1; rpl-34:mCherry strain treated with 500 μM auxin (cyan) and control (magenta). central line: median, box: interquartile ranges (IQR), whisker: ranges except extreme outliers (>1.5*IQR), individual values: crosses, extreme outliers: circles. (B) As (A), but for larval stage duration. (C) As (A), but for volume at larval molts. (D, E) As (A–C), but for raga-1:gfp:aid; myo-2p:tir1; rpl-34:mCherry strain (green) and control strain not expressing Tir1 (red), both treated maternally with 500 μM auxin. For each condition a total of at least n ≥ 150 individuals were measured on at least 2 days. *** indicate p < 10−10 (Wilcoxon rank sum test). See S2 Table for precise sample size and p-values. See S5 Data. (TIF) [file pbio.3003692.s006.tif]

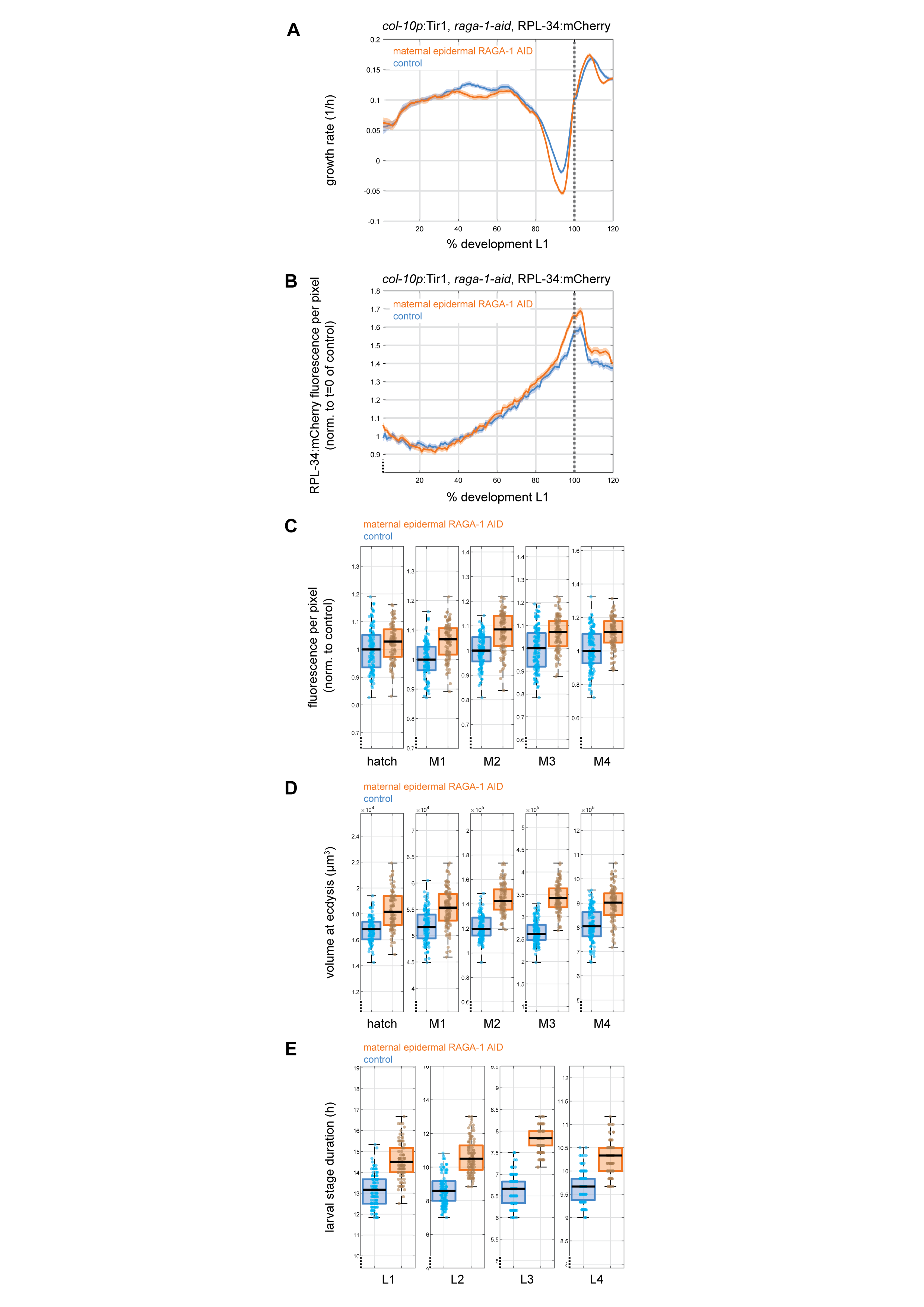

Supplement: S7 Fig — (A) Growth rate of progeny with and without depletion of maternal RAGA-1 in the epidermis. A strain expressing Tir1 under the col-10 promoter was compared to a strain not expressing Tir1. Both strains were maternally treated with 500μM auxin. Individual trajectories were aligned at hatch point and M1 and re-scaled before averaging. Range between 100% and 120% represents the beginning of L2. Solid lines: mean, shaded regions: 95% confidence interval. (B) As (A), but for RPL-34:mCherry fluorescence (intensity per pixel normalized to control). (C–E) fluorescence at hatch/ecdysis, volume at hatch/ecdysis, and larval stage duration. central line: median, box: interquartile ranges (IQR), whisker: ranges except extreme outliers (>1.5*IQR), individual values: crosses, extreme outliers were omitted from display. For each condition a total of at least n ≥ 99 individuals were measured on 2 separate days. Delay in development of col-10p:Tir strain compared to control, occurring independent of RPL-34:mCherry changes, is likely due to leaky activity of Tir1 in the absence of auxin in this strain. See S5 Data. (TIF) [file pbio.3003692.s007.tif]

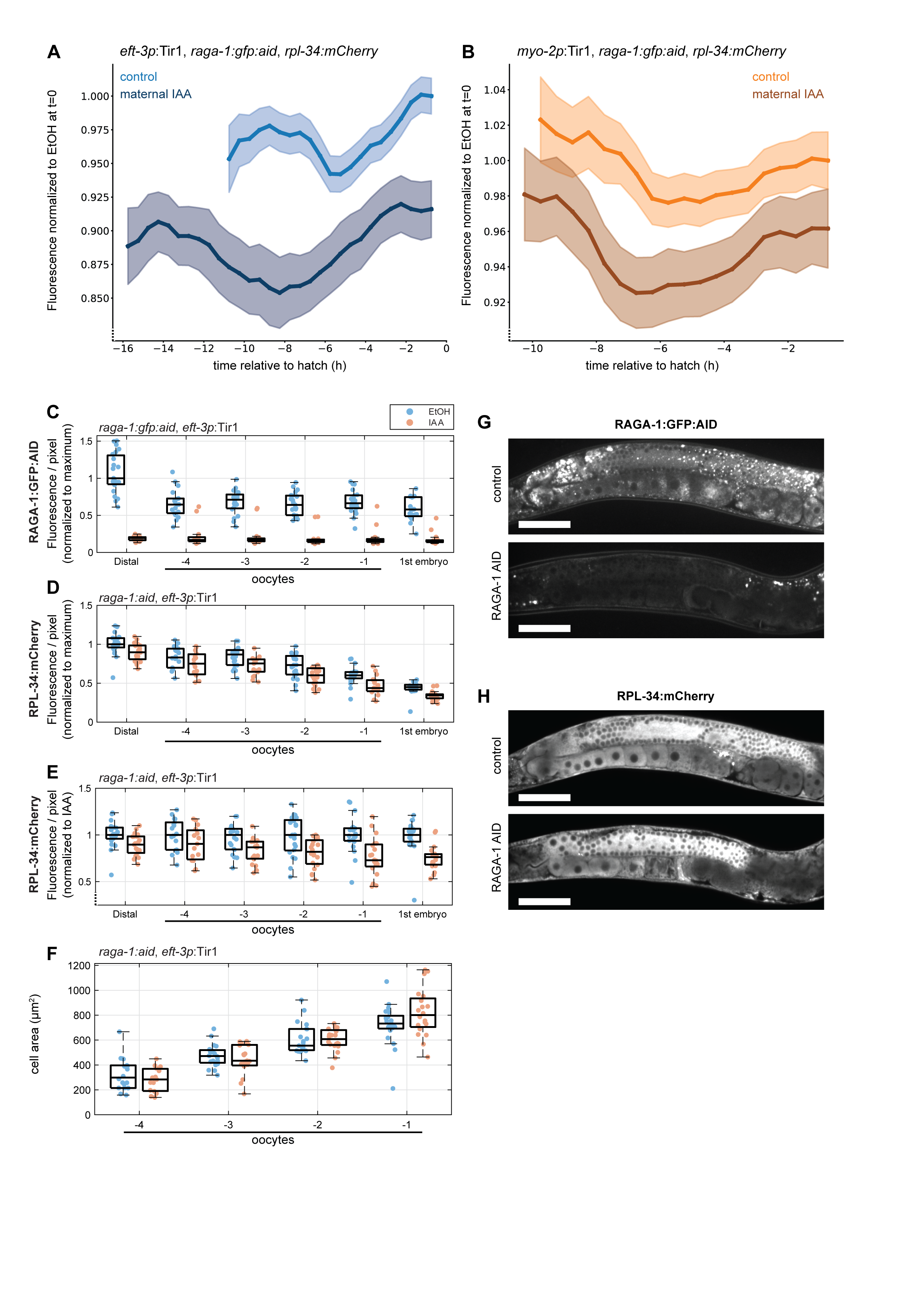

Supplement: S8 Fig — (A) Concentration of RPL-34:mCherry in embryonic progeny of RAGA-1 depleted (dark, 500 μM auxin) and control (light, 0 μM auxin) mothers expressing Tir1 under the ubiquitous eft-3p promoter. Embryos were imaged at 10-minute intervals and individual trajectories were aligned to the time point of hatching (t = 0) prior to averaging. Shaded areas indicate 95% CI of the mean. n (control) = 87 individuals, n (maternal IAA) = 55 individuals, measured on 3 different days. (B) As (A), but for strain expressing Tir1 under the pharynx-specific myo-2p promoter. n (control) = 55 individuals, n (maternal IAA) = 46 individuals, measured on 3 different days. (C) RAGA-1-GFP-AID fluorescence intensity per pixel in the germline of RAGA-1 depleted mothers (red, 500 μM auxin) and control (blue, 0 μM auxin). Distal = most distal region of the germline (first 10 rows of nuclei), −4 to −1 = oocytes at indicated distance away from spermatheca. 1st embryo = egg positioned immediately after the spermatheca. Cytoplasmic fluorescence of oocytes was measured at the central focal plane of the nucleus, omitting signal from the nuclear area. Fluorescence is normalized to the median of the most highly expressed region of control conditions. RAGA-1 is significantly depleted in the entire germline (p < 10−5 for all regions, one-sided rank sum test). central line: median, box: interquartile ranges (IQR), whisker: ranges except extreme outliers (>1.5*IQR), individual values: circles. n > 15 for all conditions and regions. (D) As (C), but for RPL-34:mCherry. RPL-34:mCherry is significantly depleted in all regions of the germline (p from distal to 1st embryo: 0.002, 0.048, 0.002, 0.001, 2*10−4, 2*10−5, one-sided rank sum test). (E) As (D) but normalized to median of respective control for each region. Relative effect size of auxin treatment increases significantly from the distal end towards proximal regions (Spearman correlation coefficient = −0.34, p-value one-sided test = 10−4). (F) Same as (D), but f [file pbio.3003692.s008.tif]

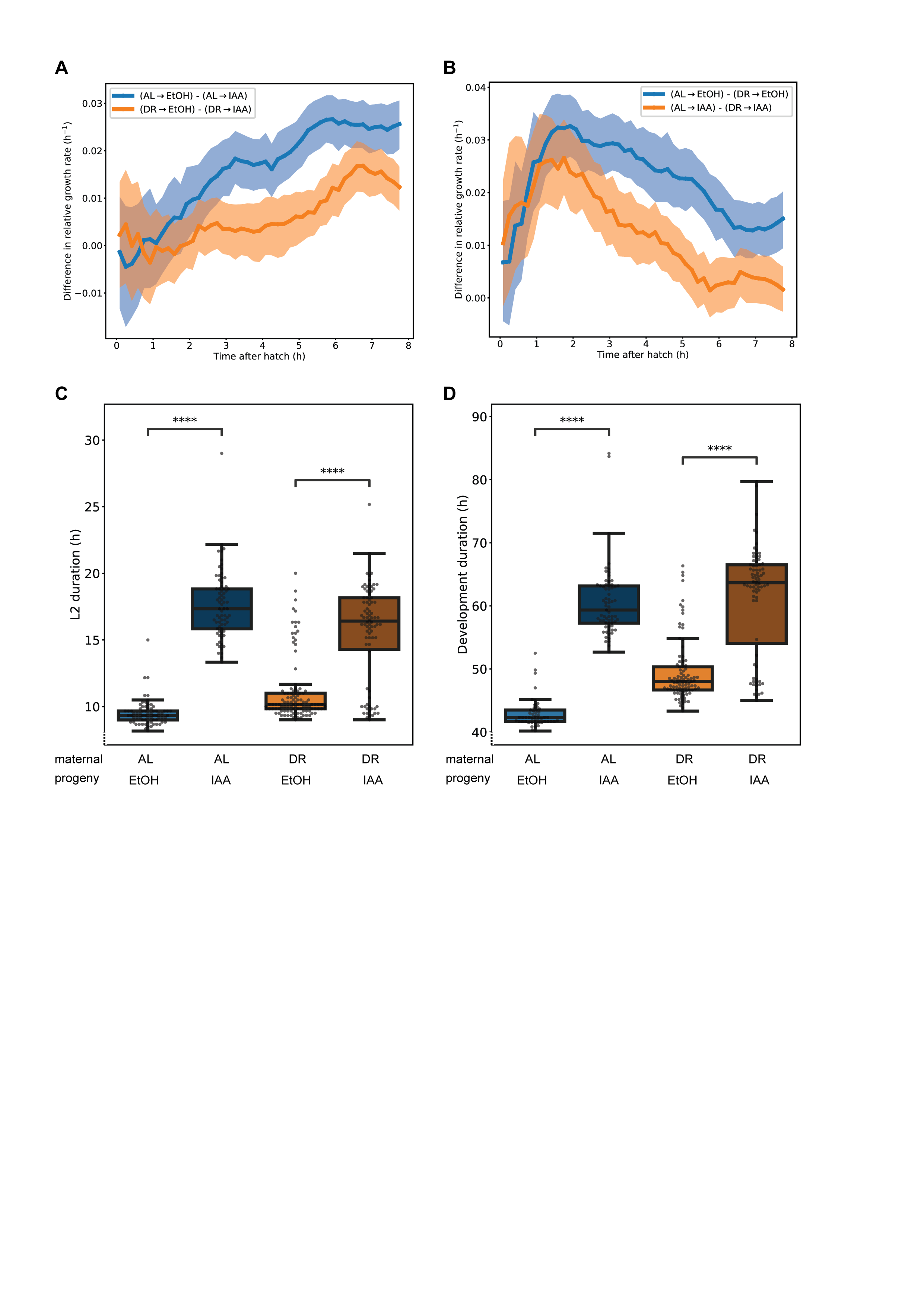

Supplement: S9 Fig — (A) Progeny of AL fed (blue) and DR mothers (orange) were grown with or without ubiquitous RAGA-1 AID (eft-3p:Tir1) in agarose chambers with abundant food. The difference in the relative (volume-specific) growth rate between progeny exposed to 500μM auxin or to vehicle control is plotted as a function of time after hatching. Shaded area indicates 95% CI. For each condition, a total of at least n ≥ 49 individuals were measured on 3 different days. (B) As (A), but for the growth rate disparity between DR and AL progeny under RAGA-1 AID and control conditions. (C) L2 duration of eft-3p:tir1, raga-1-aid animals with indicated maternal and progeny treatments. (D) As (C), but for total duration of development. **** p-value < 10−10, (Wilcoxon rank sum test). For precise p-values for each comparison, see S2 Table, S5 Data. (TIF) [file pbio.3003692.s009.tif]

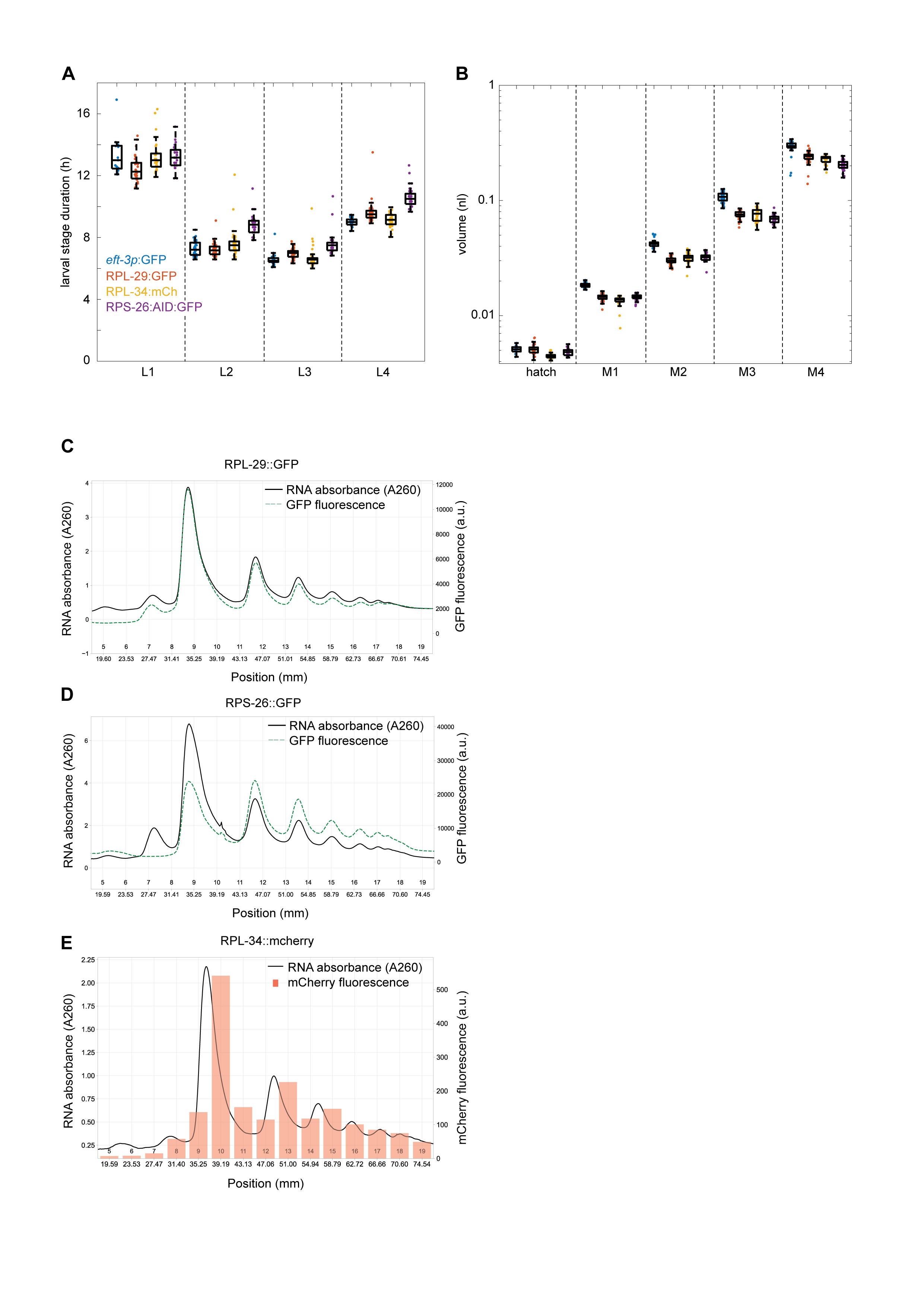

Supplement: S10 Fig — (A) Quantification of larval stage durations of indicated strains in agarose chambers. Fluorescently tagged strains develop at near normal speed compared to a strain expression free GFP under control of the ubiquitous eft-3 promoter, and similar to developmental durations measured on standard agarose plates. number of individuals n = 13, 35, 29, 29, 40, 54, 45, 44, 40, 54, 45, 44, 40, 54, 39, 44 (from left to right) measured on 1 day (B) As (A), but for volumes at larval molts. Fluorescent ribosomal protein tagging causes a slightly decreased volume. (C–E) Polysome profiles of indicated strains as L4 animals feeding ad libitum. GFP fluorescence was measured together with RNA absorbance throughout the sucrose gradient. mCherry fluorescence was measured in collected fractions using a fluorescence plate reader. Data shows that all fluorescently tagged proteins localize to translationally active polysomal ribosomes. See S5 Data. (TIF) [file pbio.3003692.s010.tif]

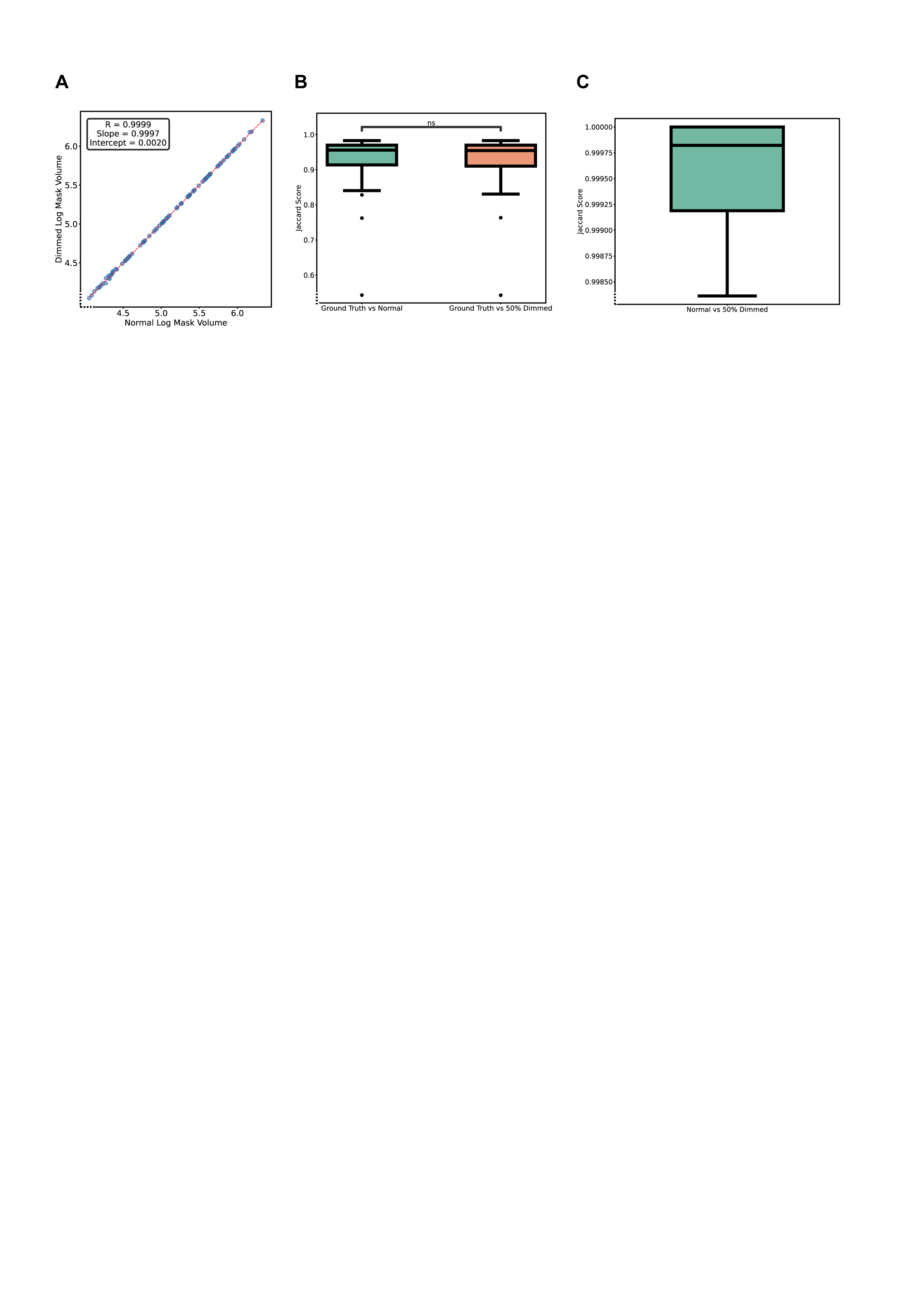

Supplement: S11 Fig — (A) 100 images of animals were computationally dimmed by 50%. Dimmed and undimmed image sets were analyzed by the same algorithm using edge detection as described in Methods. No significant effect of fluorescence intensity on volume estimation is detected. (B) Jaccard Score of segmentation masks obtained from dimmed and undimmed images compared to manually curated ground truth. No significant decrease in quality is observed after dimming. (C) Jaccard Score of Segmentation performed on dimmed images, using segmentation masks of undimmed image as ground truth. Values near 1 (=perfect identify) show negligible effect of dimming on segmentation accuracy. See S5 Data. (TIF) [file pbio.3003692.s011.tif]
