# Supplementary material for: Dietary restriction shapes intergenerational ribosome abundance and early growth of Caenorhabditis elegans offspring
Source: PLoS Biol. 2026 Apr 9;24(4):e3003692. doi: 10.1371/journal.pbio.3003692 (PMC13065021; doi:10.1371/journal.pbio.3003692)
Supplement: Attachment — Submitted filename: response_to_reviewers.pdf [file pbio.3003692.s021.pdf]

Revision of manuscript PBIOLOGY-D-25-00735R1: Response to reviewers
(Pradhan et al., Intergenerational control of ribosomes under dietary restriction)

Reviewer #1

The manuscript describes the intergenerational effect of dietary restiction on progeny growth and discovers that an observed downregulation of ribosomes in the progeny is largely sufficient for recapitulating this early growth impairment. The authors find that depletion of maternal somatic ribosomes is sufficient to induce a depletion in the progeny, and that maternal somatic depletion of the mTORc1 subunit RAGA-1 was also sufficient to induce an intergenerational downregulation of progeny ribosomes.

This manuscript is clear and well-written and represents a carefully executed body of work which definitely merits publication. It has likely relevance outside of the model organism in which the work was carried out and serves as an important example of the ways in which broad material transfers from mothers to their progeny can underlie intergenerational phenotypes, although they are often overlooked in favour of more obviously 'information-encoding' modes of inheritance such as small RNAs, as the authors highlight in the Discussion.

We thank this reviewer for this endorsement of our work.

That said, there are a couple of broad conceptual issues which the authors might be able to address either experimentally or by further discussion, in addition to some formatting and/or typological errors or ambiguities.

We are thankful for these insightful suggestions, which helped us to further improve our manuscript.

CONCEPTUAL:

1) The authors speculate in the Discussion in lines 381-387 and in their concluding lines (406-407) about the likelihood that this ribosomal downregulation may be adaptive or at least not costly for the progeny under expected conditions:

381-387: 'such a reduction is not expected to negatively impact progeny growth and fitness if dietary restriction continues across generations.'

406:407: 'Our work highlights the allocation of proteome resources to ribosomes as a new mechanism for the intergenerational transmission of phenotypic plasticity that may prepare progeny for environmental conditions that they are most likely to encounter.'

It strikes me that the experimental systems that the authors describe should be able to address these conjectures. In particular it should be possible to quantify the growth disparity of DR vs AL progeny under DR conditions, not only under AL conditions. If 'such a reduction is not expected to negatively impact progeny growth and fitness if dietary restriction continues across generations', then that predicts that this growth disparity should no longer be evident or should be negligible under continued DR conditions. Can this be tested?

We thank the reviewer for this suggestion. In response, we experimentally tested how the intergenerational ribosome effects depend on the progeny's ribosome demand. While continuous dietary restriction (DR) cannot be implemented in our sealed agarose chambers

(see note below), we used somatic RAGA-1 depletion as a well-validated proxy for reduced ribosome demand.

We find that the growth disparity between RAGA-1 AID and control conditions is significantly reduced in progeny of DR compared to *ad libitum* fed (AL) mothers, and that RAGA-1 AID causes a weaker developmental delay in DR progeny than in AL progeny, with the largest effect during the L2 stage. These data support the reviewer's prediction that reduced ribosome levels are less detrimental under conditions of low ribosome demand. We now include these results in Fig. 4E-H and Fig. S9.

In addition to reduced ribosome expression, DR progeny are also smaller. The developmental delay resulting from this size reduction occurs independently of the growth conditions of the progeny, as animals hatching with a smaller size require more time to reach the adult size. In the revised Discussion (ln. 431), we now state that the growth disparity between DR and AL progeny is expected to be smaller under conditions of slow progeny growth, but not necessarily completely abolished. In addition, we have adjusted the language throughout the manuscript to describe the observed growth pattern without explicitly speculating on an adaptive function (e.g., ln. 439: "[Our work] highlights the allocation of proteome resources to ribosomes as a mechanism of intergenerational plasticity that modulates early progeny growth as a function of the post-hatching demand for protein synthesis capacity.").

Technical note: Our agarose-based imaging system requires bulk feeding of bacteria loaded at start within afterwards sealed chambers. At low food concentrations, larvae exhaust the available food before L1 completion, making sustained DR conditions infeasible and irreproducible. While microfluidic systems allow food replenishment, currently available designs are incompatible with both high-throughput embryo loading and/or the imaging precision required here. We now mention this explicitly in the discussion (ln. 434)

2) The authors suppose in the Discussion that the inherited 'signal' may be ribosomes deposited in the oocyte that were synthesised in the proximal germline. They show that ribosome depletion in the maternal soma and the distal germline do not recapitulate the intergenerational growth phenotype but they do not perturb ribogenesis in the proximal gonad or directly examine the response of ribogenesis in the proximal gonad to somatic RAGA-1 disruption. It would be nice to have some more evidence that the 'signal' involves ribogenesis specifically in the proximal gonad.

While specific perturbation of ribosome biogenesis in the proximal germline is currently not technically feasible (proximal germline–restricted Tir1 drivers are not available and would likely affect early embryos as well), we directly addressed whether differences in progeny ribosome levels arise from altered embryonic production or from maternal deposition by two new experiments: First, we performed live imaging of RPL-34:mCherry levels during embryogenesis. Second, we measured the RPL-34:mCherry concentration in oocytes and the distal germline.

To assess embryonic ribosome production, we used live imaging to quantify the increase in ribosomal signal during embryogenesis. If the reduced ribosome content in maternally RAGA-1 AID-treated animals resulted from decreased embryonic synthesis, auxin treatment would be expected to reduce the slope of increase in ribosomal protein signal. However, no such difference was observed in progeny of mothers with ubiquitous or pharyngeal RAGA-1 depletion. Instead, the difference between control and auxin treated conditions remained nearly constant throughout embryonic development. These data support the conclusion that the observed

intergenerational differences primarily reflect the ribosomes deposited into the oocyte or early embryo, rather than embryonic ribosome synthesis.

In addition, we quantified RPL-34:mCherry in the distal germline, in oocytes of the maternal germline, and in the first embryo after fertilization in ubiquitously RAGA-1-depleted mothers, and matched controls. We observe a slight reduction in the RPL-34:mCherry concentration in the germline upon RAGA-1 AID, with a more pronounced reduction in more proximal oocytes. Notably, independent of RAGA-1 AID treatment, the RPL-34:mCherry concentration decreases as oocytes approached the spermatheca, likely reflecting rapid oocyte growth not matched by proportional ribosome synthesis. This decline was more pronounced in RAGA-1 AID animals, consistent with a role of ribosome regulation in the proximal gonad in determining progeny ribosome levels.

These new data are shown in Fig. S8. We also clarified in the revised Results and Discussion that potential mechanistic differences operating in different germline sub-regions remain to be determined (ln. 277, ln. 416).

We agree with the reviewer that while direct depletion of RPS-26:AID:GFP using germline-driven Tir1 transgenes established that modest reductions in progeny ribosomal protein levels are sufficient to impair growth, these experiments did not conclusively address the spatially resolved regulation of ribosome abundance within the germline. Our new quantitative, region-specific measurements provide a more precise spatial analysis than our previous coarse analysis of ribosomal protein distribution along the proximal–distal germline axis upon RPS-26:GFP:AID. We have removed this earlier analysis to improve clarity and to focus on the more informative data presented here.

TYPOGRAPHICAL ISSUES

- Several places compound adjectives should be hyphenated. e.g.

37 near-linear

Done.

120 membrane-bound

Done.

311 somatically-reduced

The relevant sentence has been removed from the revised text.

374 long-term

The relevant sentence has been removed from the revised text.

- line 43 under conditions of DR

Done.

- line 73 'progeny of DR animals WERE exposed'

Done.

- line 93 'Downregulated proteins were enriched for translational machinery, particularly in ribosomes^ and vitellogenins (yolk proteins) while upregulated proteins were...'

Comma needed at ^? Yolk proteins are not translational machinery

Done.

- lines 89, 448. Numbers to be written as 1,000 not 1'000

Done.

- Fig S1B: typo 'Upwnregulated'

Done.

AMBIGUITY

line 86 - ambigious phrasing 'Dietary restriction (DR) was imposed by a five-fold food dilution compared to ad libitum (AL) feeding (2*108 vs. 109 cfu/ml), which slowed down development from three to five days per generation (Fig. 1A).'. Although I understand the sense of it and it is clear with reference to Fig 1A, it could be read as that DR slowed down development 3-5 days per generation, which isn't what they mean. Rephrase to avoid any potential confusion.

This sentence (no in line 89) was adjusted and reads now: "Dietary restriction (DR) was imposed by a five-fold food dilution compared to *ad libitum* (AL) feeding ($2*10^8$ vs. 10^9 cfu/ml). Food dilution slowed down development to adulthood by 2 days per generation (changing the generation time from 3 days for AL to 5 days for DR fed animals) (Fig. 1A, Fig. S1A)."

Fig S1A is a strange figure that contains very little information; in the end the information contained could easily be reported in the text in two figures (6454 proteins for P0, 6432 for F1). Also it seems odd that the number of proteins detected is exactly the same for each sample within each generation (but slightly different across generations). Is this correct?

We agree and have therefore removed Fig. S1A, instead reporting the number of identified proteins directly in the main text (ln. 96). We also added a sentence to the Methods (ln. 509) to clarify the design of the TMT experiment.

The identical number of detected proteins across replicates within each generation is expected for TMT-based proteomics. In this approach, samples are multiplexed and peptide identification is performed prior to sample assignment by mass tags, resulting in identical protein identification across samples within a single TMT run and improved quantification of relative changes compared to label-free methods.

In our experiments, three biological replicates of AL and DR conditions were combined into a single TMT6 run for each generation, with separate TMT6 experiments performed for G0 and G1. Consequently, the number of identified proteins is identical across conditions and replicates within each generation but differs slightly between generations. Importantly, fold changes were calculated only within each generation, i.e., within the same TMT experiment.

line 386; use plain English in the Discussion when referring to the DR to AL shift. Here to make the broader point it is better to avoid the jargon and abbreviations.

Done.

TERMINOLOGY

line 54: 'called intergenerational inheritance when lasting for only one generation, or transgenerational inheritance when lasting for longer'. While the point is not very relevant to the current paper this isn't always correct for all organisms. See Heard & Martienssen 2014 [PMID 24679529] Fig 1 for nuance. Some would argue even in C. elegans that a 2 generation effect might be considered intergenerational (as the germ cells may already be present in progeny in utero when their grandparents are exposed to an environmental stimulus during adulthood). 'Parental effects' would be limited more specifically to one generation.

We removed this distinction from the text, as we agree this definition is tangential to the current manuscript. The section reads now: “Despite this barrier, the parental environment can have a pronounced impact on the physiology of the offspring (9), which in some cases can last over many generations (10). Examples include maternal effects on resistance to pathogens (11), osmotic stress (12), temperature (13), or starvation (14).” (ln. 54)

At the risk of being pedantic, F1 stand for Filial generation and is derived from a genetic cross. As that is not the case here, P0 / F1 might be better expressed as G (for generation) 0 / G1

We agree and follow this advice in the revised manuscript, replacing F1 with G1 and P0 with G0 throughout the text and figures.

lines 160/209/206/270: the authors several times use m e.g. m = 3. As this is repeated, I assume this is deliberate, I guess to distinguish from the reported n (which does stand for 'number' of individuals). Nonetheless this convention of using m was unfamiliar and unintuitive to me, as it is not obvious what 'm' stands for.

Non-conventional variable use has been removed throughout the manuscript.

Reviewer #2:

In this study, the authors combine proteomics, genetics, and live imaging in C. elegans to uncover an intergenerational regulatory mechanism in which maternal dietary restriction leads to reduced ribosomal protein levels in offspring. While most components of the proteome are reset between generations, ribosomal proteins remain reduced in progeny, delaying their growth until ribosome levels normalize. The authors show that this effect is mediated by mTORC1 signalling in the maternal soma, as soma-specific depletion of RAGA-1 mimics dietary restriction. These findings suggest that maternal mTORC1 activity communicates across the soma-germline barrier to program ribosomal provisioning in offspring, revealing a novel axis of intergenerational regulation.

This study presents a compelling conceptual advance and will be of interest to a wide audience. The data is generally well-analyzed. However, there are major points that should be addressed to support the conclusions made.

We thank the reviewer for the thoughtful comments, which have helped us to substantially strengthen our manuscript. We address each point in detail below.

Major Comments:

Somatic specificity of mTORC1 depletion via eft-3p::TIR1 requires further validation.

The central conclusion that maternal somatic mTORC1 activity modulates progeny ribosome content hinges on the assumption that eft-3p::TIR1-driven RAGA-1 degradation is restricted to somatic tissues. While eft-3 is typically used as a pan-somatic promoter, it is derived from a translation elongation factor and has been reported to drive detectable expression in the germline under some conditions—especially over long auxin exposures or high concentrations (e.g., 500 µM to 1 mM for >18 hours). In this study, RAGA-1::GFP depletion was assessed after 26 hours of 500 µM auxin, and while soma-specific reduction is visually presented (Fig. 4A), quantification of RAGA-1::GFP signal in the germline versus soma would be essential to convincingly demonstrate specificity. This could be done either in whole animals or dissected gonads, using the same imaging conditions.

Alternatively (or additionally), the authors could support this claim by using a more germline-restrictive strategy, either by repeating the experiment with a validated somatic-only TIR1 driver such as rgef-1, myo-3, dpy-7, or col-10 (as described in Ashley et al., Genetics 2021, PMID: 33677541), or by employing a mosaic approach as described in Artiles et al. (Dev Cell, 2019, PMID: 30799227), which allows for tissue segregation in F1. While the mosaic analysis is technically more demanding, even a limited cohort of 5-10 F1s with clear soma vs germline separation would significantly strengthen the conclusion that the effect is truly somatic in origin.

We thank the reviewer for raising this important point. We agree that *eft-3p*:Tir1 can show variable germline activity, depending on strains-specific transgene silencing and duration of auxin exposure, which we now show in the revised manuscript (Fig. S8C). We have revised the manuscript accordingly and do not refer to *eft-3p*:Tir1 anymore as “soma-specific”, but instead as “maternal RAGA-1 depletion”.

To directly demonstrate that maternal somatic mTORC1 activity can influence progeny ribosomal protein levels, we performed the second suggested experimental approach using a validated soma-restricted TIR1 driver, which confirmed our previous conclusion, and is now shown in Fig. 4C, D and Fig. S6D-F.

The additional experiments performed are described in detail below.

1. Validation using a germline-exclusive somatic depletion strategy

Following the reviewer’s recommendation, we used a validated somatic-only driver (*myo-2p*::Tir1) to degrade RAGA-1 specifically in the pharynx. This depletion significantly reduced progeny ribosomal protein levels and delayed larval development, consistent with the phenotype of dietarily restricted mothers. These data provide direct evidence that perturbing maternal somatic mTORC1 activity is sufficient to drive the intergenerational effect. Results are now shown in Fig. 4C, D, and Fig. S6D-F. Additionally, we added a paragraph to the Discussion (ln. 410) on possible mechanisms for this cross-tissue effect.

In contrast to pharyngeal-specific RAGA-1 depletion, epidermal-specific RAGA-1 depletion via *col-10p*:Tir1 reduced maternal growth, but did not reduce progeny ribosomal proteins, supporting our conclusion that parental growth defects alone do not explain the progeny phenotypes. This data is shown in Fig. S7.

Since the P1 lineage also gives rise to somatic tissues, such that mosaic manipulations of the AB/P1 lineages do not generate a complete soma-germline separation, we did not pursue this

elegant, yet technically demanding approach. Obtaining sufficiently large cohort sizes to provide the statistical power needed for the expected effect sizes observed would be very challenging.

We also note that other suggested Tir1 drivers (e.g., *myo-3p*:Tir1) did not significantly reduce maternal growth when used to deplete RAGA-1 by AID. Since we cannot distinguish if this lack of effect is of biological or technical nature, we decided not to include these drivers in the revised manuscript.

2. Quantitative assessment of RAGA-1 depletion using *eft-3p*:Tir1

As suggested by the reviewer, we quantified germline RAGA-1:GFP levels under *eft-3p*:Tir1-mediated AID and identified significant germline depletion of RAGA-1:GFP in the strain used for RPL-34:mCherry measurements (shown in new Figs. S8C, G). The variable degree of soma-specificity of *eft-3p*::Tir1 transgenes is consistent with previously reported strain-dependent and temporally fluctuating variability in germline silencing of *eft-3p*-driven transgenes (PMID: 27374334).

Because *eft-3p*:Tir1 does not reliably restrict degradation to the soma, we now refer to this condition as "maternal RAGA-1 depletion" rather than a "soma-specific" manipulation. We rely on the validated somatic-only driver *myo-2p*:Tir1 to support our conclusions regarding soma-specificity.

Ribosome concentration measurements rely on tagged RPs, here functional validation is needed. The conclusion that ribosomal protein levels are reduced in progeny is primarily based on fluorescent intensity measurements of RPL-34::mCherry and RPL-29::GFP. However, these are transgene insertions, and it remains unclear whether the tagged proteins are functionally incorporated into mature ribosomes or able to complement loss of function alleles. Without such validation, fluorescence intensity may not fully reflect functional ribosome concentration but instead also detect free unassembled ribosomal proteins. At minimum, the authors should clarify in the Methods whether the alleles are functional (e.g., rescue data, absence of growth defects), and whether incorporation into ribosomes has been previously demonstrated (polysome analysis, or prior publications). If this information is not available, the conclusions regarding "ribosome concentration" should be stated more conservatively as "fluorescence levels of tagged ribosomal proteins."

We have addressed this comment by further validation experiments of functionality, and by textual edits to better explain the genetic nature of the fluorescently tagged ribosomal proteins, and by more precise statements of the results obtained.

<u>Genetic nature of the tags:</u> All fluorescently tagged ribosomal proteins were generated by CRISPR/Cas9 and were inserted at the endogenous locus, fully replacing the untagged wild-type copy. We now clarify this design explicitly in the Results (ln. 160) and in the Methods (ln. 445ff).

<u>Validation:</u> To directly address functional incorporation and any potential growth effects associated with the protein tags, we have added new validation data shown in Supplemental Figure S10:

1. Polysome profiling shows that all there fluorescently tagged ribosomal proteins incorporate into actively translating ribosomes.

2. Developmental timing does not, or only minimally, change in the tagged strains.
3. We observe a minor body size reduction in tagged RP strains. Importantly, however, all comparisons were performed within matched genetic backgrounds carrying the same tag. These controls allow us to assign the effects to maternal diet or RAGA-1 depletion, rather than the fluorescent tags used.

We further emphasize that our conclusions are supported by multiple orthogonal controls:

1. Mass spectrometry in untagged backgrounds confirms that dietary restriction reduces ribosomal protein abundance.
2. Three different ribosomal proteins, tagged with two distinct fluorophores, all show consistent intergenerational effects.
3. Control proteins not associated with ribosomes do not exhibit intergenerational changes.

Precision of statements: Our additional experiments show that fluorescently tagged ribosomes are functional and incorporated into polysomes. Yet, we agree with the reviewer that referring to “ribosomal proteins” is a more precise than to “ribosomes”. We have adjusted the wording throughout the text for improved clarity and precision.

In Figure 3, degradation of RPS-26 in the germline (using sun-1p::TIR1) results in reduced progeny growth and ribosome concentration, whereas depletion of RPL-22 in the soma (eft-3p::TIR1) or distal germline (gld-1p::TIR1) does not. However, no tissue matched depletion of the same RP is shown so it's unclear whether the difference is due to tissue specificity or intrinsic differences between RPs.

We agree that using different ribosomal proteins in the original comparison made it difficult to distinguish tissue-specific effects from potential intrinsic differences between RPs or other strain-specific effects. To address this, we have now performed maternal RPS-26:AID:GFP depletion under *eft-3p*::TIR1, ensuring that all tissue-specific and ubiquitous perturbations use the same RP. These new data are included in Supplemental Figure S3A-E.

The *eft-3p*:Tir1-mediated depletion of RPS-26:AID:GFP caused a significant reduction in progeny ribosomal protein levels that is stronger than what we observed for RPL-34:mCherry after RPL-22 depletion. We attribute these quantitative differences between these strains most plausibly to differences in the Tir1 expression level caused by strain-specific germline silencing of *eft-3p*:Tir1 (see our response to comment #1 above), although we cannot definitively exclude other additionally contributing factors.

Because RPL-22:AID is not fluorescently tagged, we cannot directly quantify its depletion, and its inclusion made comparative interpretation more difficult. To improve clarity and focus, we have therefore removed all RPL-22:AID data from the revised manuscript and now present a unified dataset using RPS-26:AID:GFP for all tissue-specific comparisons, in line with this reviewer’s and reviewer #3’s suggestion.

Importantly, the central conclusion remains fully supported and unchanged: Reducing ribosomal protein levels of the L1 progeny at hatching reduces their initial growth rate.

We also provide additional evidence in Supplemental Figure S7 that reduced maternal growth alone is not sufficient to lower progeny ribosome levels: epidermal RAGA-1 depletion substantially slows maternal growth but leaves progeny ribosomal protein levels unchanged.

What is the correlation between dietary restriction (DR) and the mTORC1 pathway in the intergenerational regulation of ribosomes and growth rate?

In our original submission, we demonstrated that maternal dietary restriction and maternal mTORC1 inhibition produce highly correlated phenotypic outcomes in progeny. Specifically, both conditions result in: Reduced growth rate, smaller size, and reduced ribosome levels at hatching, and prolonged L1 duration.

Does dietary restriction act through mTORC1?

We interpret the comment as referring specifically to epistasis analysis between DR and RAGA-1 depletion.

We now provide new data in Fig. 4E-H and in Fig S9, showing that reducing mTORC1 activity in progeny (via RAGA-1 depletion) diminishes the growth difference between AL- and DR-derived progeny. This intergenerational epistatic interaction is consistent with both manipulations lowering the ribosome requirement for growth.

We did not perform maternal epistasis analysis (i.e., applying DR to mothers already depleted of RAGA-1) because classic binary epistasis logic does not apply in this system:

Epistasis interpretation requires fully penetrant, non-redundant loss-of-function in the downstream component (Huang & Sternberg 2006, wormbook.org; Avery & Wasserman 1992, PMID: 1365397). However, mTORC1 activity cannot be fully removed since complete loss (e.g., raptor/*daf-15* mutants) is lethal, and AID-based RAGA-1 depletion leaves residual mTORC1 signalling. Similarly, dietary restriction is inherently quantitative, and not a clean *null* phenotype. Therefore, the standard "mutation A masks the phenotype of mutation B" framework would not yield a definitive conclusion about pathway ordering in this case.

We now make explicit in the revised manuscript that, while RAGA-1 depletion recapitulates key DR phenotypes, and DR effects require mTORC1 activity in the progeny, we do not claim that all DR responses are mediated exclusively via mTORC1 (ln. 321).

Depletion of RAGA-1 in the maternal soma (*eft-3p*:Tir1) phenocopies the effects of maternal dietary restriction. What are the phenotypes resulting from RAGA-1 depletion in the proximal or distal germline?

We appreciate the reviewer's interest in germline-specific mTORC1 function. However, given our new data demonstrating that *eft-3p*:Tir1 also drives depletion of RAGA-1 in the germline and that pharynx-specific RAGA-1 depletion using *myo-2p*:Tir1 similarly reduces progeny ribosomal proteins levels, we believe that additional germline-restricted RAGA-1 depletion experiments would not add to further support the key claim that somatic mTORC1 activity can influence progeny ribosome provisioning.

Technically, generating new strains with degradation of RAGA-1 restricted to the germline would require multiple new allele combinations and long-term live-imaging across numerous replicates, which would together demand several months of work. Importantly, based on our updated findings, these experiments would not substantially strengthen our central conclusion.

Instead, we have taken a functionally equivalent approach that addresses both soma-driven and germline-driven mechanisms:

1. Somatic RAGA-1 depletion by *myo-2p*:Tir1 (pharynx) reduces progeny ribosome levels and delays development. This demonstrates that soma-restricted reduction of mTORC1 can reduce progeny ribosomes.
2. *col-10p*:Tir1 (epidermis) strongly slows maternal growth but does *not* affect progeny ribosomes. This demonstrates that reduced maternal growth alone does not drive intergenerational effects.
3. Germline ribosome depletion of ribosomes by *sun-1p*:Tir1 reduces progeny ribosome levels and growth. This data confirms that altering ribosome provisioning to offspring affects progeny phenotypes.
4. Ubiquitous RAGA-1 depletion by *eft-3p*:Tir1significantly reduces RAGA-1 in the germline and RPL-34:mCherry levels in the oocytes and reduces progeny growth.
5. Ubiquitous or pharynx-specific maternal RAGA-1 depletion does not alter ribosomal protein production during embryogenesis.

Together, these data strongly support the tissue-specific logic that somatic mTORC1 modulation can alter ribosome depositioning through the germline, and ribosome depositioning alters progeny growth.

Finally, could mTORC1 activation during DR rescue or suppress ribosome concentrations and growth rate? Evidence or elaboration on this would greatly improve the manuscript.

We thank the reviewer for this insightful suggestion. To address it, we analyzed a *raga-1* gain-of-function (gf) allele, predicted to increase mTORC1 activity due to a reducing GTPase activity (PMID: 39300311).

We find that hyperactivation of RAGA-1 partially suppresses the intergenerational DR phenotype with respect to the L1 duration and volume at hatching. However, ribosomal protein levels in progeny remain influenced by maternal diet in the *raga-1(gf)* background. Thus, mTORC1 hyperactivation reduces the phenotypic impact of maternal DR on growth dynamics but does not eliminate the diet-dependent maternal control of ribosomal protein levels. This remaining regulation of ribosomal proteins may be due to retained regulatory potential of the *raga-1(gf)* allele, or due to an additional, *raga-1*-independent, regulation of ribosomes.

Importantly, these results agree with our previous notion that while the ribosome concentration is regulated across generations, it is not the only mechanism of intergenerational inheritance. For clarity and focus of our manuscript, and since these data neither alter our central conclusions, nor provide a definitive mechanistic dissection of upstream regulation, we provide these results as a figure to reviewers below but did not include these data in the revised manuscript.

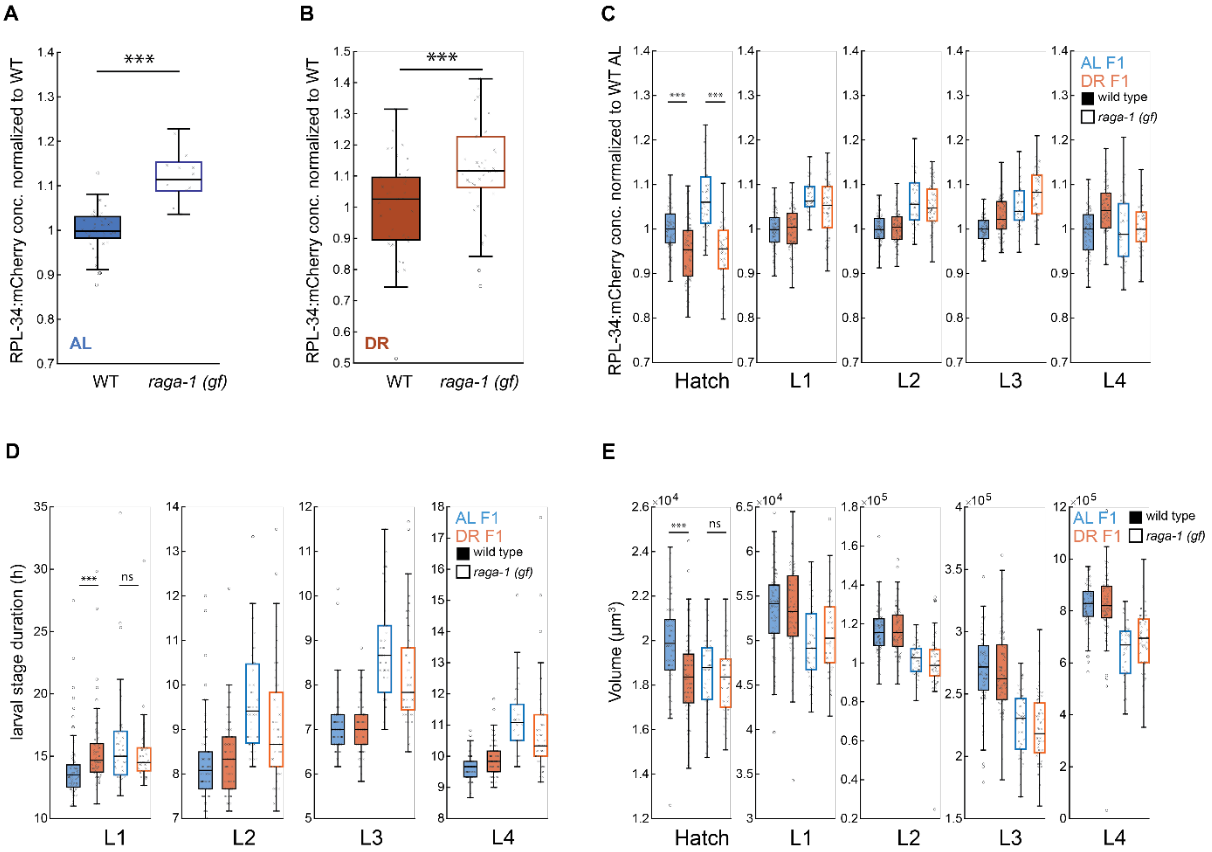

Effect of *raga-1(gain-of-function)* on intergenerational ribosomal protein control

A strain carrying a mutation in the GTPAse domain of RAGA-1, which is reported to hyperactivate mTORC1 (PMID: 39300311) was introduced into a strain endogenously tagged with mCherry at the *rpl-34* locus. The parental generation was exposed to dietary restriction and progeny growth and ribosome expression was measured in agarose chambers with abundant food.

(A, B) RPL-34:mCherry fluorescence intensity for wild type and *raga-1(gf)* in parental generation as young adults. The *raga-1(gf)* mutation enhanced RPL-34:mCherry expression under AL and under DR. **(C-E)** RPL-34:mCherry concentration, larval stage duration, and volume at hatching and larval molts. The larval stage duration and volume at hatching are not significantly altered by maternal DR in *raga-1(gf)* mutants, but RPL-34:mCherry level are modulated by maternal diet in mutant and wild type progeny. *** indicate $p < 10^{-5}$ (Wilcoxon rank sum test).

Minor Comments:

Line 89: Change "6'400 proteins" to "6,400 proteins" for formatting consistency.

Done.

Line 103: Define the KS test acronym before first use.

Kolmogorov Smirnov was used only once, so abbreviation (KS) was removed entirely.

Line 113 / Figure 1D: Please clarify whether GO enrichment analysis was based on significantly regulated proteins or on z-scored trends across generations. The source and method for the GO analysis should also be described (tool or database used).

GO enrichment was based on z-score trends. This is now explicitly stated in the legend of Figure 1D and in the main text (ln. 143). The tool used is now stated in the Methods (ln. 525). Proteins used as input for GO enrichment analysis are marked in source data table to Figure 1D.

Line 456: Consider updating the section header to "TMT Mass Spectrometry and Analysis" for clarity.

Done (ln. 511)

Methods section: Please include full details of auxin treatment (concentration, duration, temperature, developmental stage, etc.) for each relevant experiment.

This information is now available in a new Methods section “Auxin-inducible degradation” (ln. 633ff).

It appears that Table S1b is missing, and the list of differentially expressed genes shown in Figure 1E has not been provided.

Supplemental Table S1b was provided, but unlike Supplemental Table S1a, was part of the main Supplemental file itself. It is now also added to the corresponding Supplemental File as a separate sheet.

The volcano plots shown in Figure 1E stem from the same experiment as those shown in Figures 1B and 1C, for which differentially regulated proteins are shown in Supplemental Table S1. The data is shown again in 1E to mark ribosomal and proteasomal proteins.

The data presented in Figure 1 is available as a source data file including the list of genes used for GO term enrichment in Figure 1D. Access to full raw data is available on the provided link to the PRIDE repository.

Reviewer #3

In the manuscript "Intergenerational control of ribosomes under dietary restriction" Pradhan et al demonstrated that dietary restrictions (DR) in the parental generation lead to phenotypic changes in the offspring (F1), characterized by a slower growth rate and reduced body size during early development, specifically in the larval stage (L1). The authors then performed quantitative proteomics and P0 and then double starved F1s to reveal that ribosomal proteins were significantly reduced in both the parental generation (P0) at the young adult stage and in the F1 reexposed offspring at the L1 stage, which contributes to these phenotypes (slow growth and decreased body size). The authors also depleted certain ribosomal proteins in the P0 germline, which resulted in a phenotype similar to that of the DR mothers. Additionally, the study demonstrates that somatic RAGA-1 signaling is crucial in the parental generation, as it regulates ribosomal protein levels in the next generation (F1). Depleting somatic RAGA-1 in the P0 was sufficient to replicate the DR phenotype in F1.

The authors phrasing of this manuscript has substantial problems because they have starved the F1 worms to achieve the arrested L1 stage which means that they are starving both the parents and the children, and this is not really measuring intergenerational protein levels but whether repeated starvation is different from single starvation. This is still an interesting question but substantially different from how the authors introduce the topic, therefore the introduction, title, and conclusions need to be rewritten to reflect what was performed.

Additionally, reduced levels of maternally loaded ribosomal proteins or other vitellogenin proteins (yolk proteins) in the mothers due to DR could be the cause of the observed phenotypic changes. It is possible that DR does not directly alter ribosomal protein expression in F1, but rather results in decreased maternal ribosome loading, which leads to minor phenotypic changes in early L1 progeny. Furthermore, a previous study (PMID: 30799226) indicates that maternally loaded ribosomes are critical for early development and are sufficient for the F1 progeny to produce fully functional first-stage larvae, with no change in body length at hatch observed even when ribosomal proteins in F1 are non-functional.

We thank the reviewer for these comments. We address them below point-by-point.

<u>Phrasing of the manuscript and overnight starvation for proteomics:</u>

We agree that overnight starvation used for synchronizing L1 population could, in principle, influence ribosomal protein levels. However, our experimental design directly controls for such effects, and our conclusions do not depend on starvation-induced effects in the F1 generation, specifically:

1. All proteomics comparisons are conducted between progeny treated identically post-hatching. Thus, differences arise from maternal diet, not from conditions experienced by the progeny.
2. In addition to proteomics (Fig. 1), we conducted live-imaging measurements (Figs. 2-4), which are performed within <10 minutes of hatching in the presence of food (i.e., without starvation). The results of these experiments were fully consistent with proteomics-based measurements.
3. New data (Supplemental Fig. S8) show that ribosomal protein differences are already present in embryos, prior to any handling or starvation.

Together, these data demonstrate that the observed reduction in ribosomal proteins is not an acute response to progeny starvation and that it is caused by the maternal diet.

<u>Maternal loading:</u>

The reviewer suggests that the observed phenotype may be explained solely by altered maternal loading of ribosomes or yolk proteins. We agree that maternal provisioning of ribosomal proteins is the most parsimonious mechanism. We see this as a key advance of our manuscript: that maternal loading of core cellular machinery can impact phenotypes intergenerationally. We have revised our Discussion to more explicitly state this model (ln. 397) and added new data, showing that the RPL-34:mCherry concentration is strongly reduced in oocytes of RAGA-1 depleted mothers (Fig. S8D, E).

We agree that other proteins, such as vitellogenins are differentially loaded into progeny, depending on diet (Supplemental Table S1b), which we mention in the revised Discussion (ln. 420). However, we show that selective depletion of ribosomal proteins in the maternal germline (without dietary change) is sufficient to recapitulate the slower progeny growth. Thus, ribosome provisioning is causal for the observed growth effects, not merely correlated with changes in yolk or other maternal factors. Moreover, in new analysis, in which we reduced vitellogenin loading by RNAi of the yolk receptor *rme-2*, we find that reduced vitellogenin loading does not reduce the RPL-34:mCherry concentration in progeny (see Figure provided to reviewers below).

<u>Cited work concerning maternally loaded ribosomes (PMID: 30799226, Cenik et al. 2019):</u>

We now more extensively discuss this important study by Cenik et al. in a dedicated paragraph at the beginning of the Discussion (ln. 397, 3rd paragraph). Cenik et al. demonstrated that maternally provided ribosomes are sufficient to support embryogenesis and that an inability to produce ribosomes after hatching leads to a Cell non-autonomous larval growth arrest. While these results are fully consistent with our work, our findings do not overlap with this paper. Rather, they extend upon it by showing that maternal diet modulates the concentration of maternally provided ribosomal proteins, revealing the physiological regulation of this critical pool.

In addition to these below are some experimental suggestions that will significantly improve the manuscript.

Major points

1) Arrested L1 larvae is achieved how? This seems to be achieved by bleaching and then growing the eggs in media without food which therefore eliminates anything inherited as you are subjecting it to a second successive generation of starvation. This is a very different question than the way you have introduced the paper. The intro and the conclusions therefore need to be rewritten to reflect what you have actually done. When you try to make conclusions about comparing the P0 to the F1 this is obviously impossible to do as you are comparing apples to oranges.

Arrested L1 larvae were generated using standard L1 synchronization by bleaching gravid adults and allowing embryos to hatch overnight in the absence of food. While this procedure involves a brief post-hatching starvation period, it is applied identically to all progeny regardless of parental diet. Therefore, any differences observed among progeny animals cannot arise from L1 starvation itself, but instead reflect differences in maternal dietary history.

Consistent with this interpretation, our live-imaging experiments in agarose chambers show that reduced ribosome levels are already detectable immediately after hatching, before prolonged L1 arrest could exert effects. Importantly, in these experiments, larvae hatch in the presence of food and are imagined within less than 10 minutes after hatching. In addition, new data presented in Supplemental Fig. S8 demonstrate that these differences are evident already at the embryonic stage, prior to hatching altogether. Together, these results confirm that the observed intergenerational phenotypes do not depend on L1 starvation. We have revised the Results section to more clearly describe our experimental design and analytical framework (lines 93 and 208).

We agree that direct comparison of the proteome of progeny L1 and maternal adult animals would not be informative. Rather, we assess the effects of dietary restriction within each generation independently, normalizing each stage to its corresponding stage-matched, untreated control. We then compare the fold changes between generations. We have revised the relevant Results section to more clearly explain this analytical approach (ln. 143).

2) It would strengthen the paper to perform your auxin treatment of RPS-26 and raga-1 with or without parental DR and see if the ribosome depletion or TOR depletion further exacerbates the developmental slowing or if they are truly epistatic. Otherwise, you have two separate findings, that parental starvation + additional kid starvation leads to reduced ribosomes and that that same treatment leads to reduced growth rates.

We agree that RAGA-1 depletion and DR likely affect additional processes beyond ribosomes and discuss this in the manuscript (ln. 267, ln. 323). However, we respectfully disagree that our findings are merely correlative: in Fig. 3, we show that a direct ~12% reduction of RPS-26:GFP by AID, which matches the magnitude seen after maternal DR, is sufficient to slow growth at hatching. Thus, reduced ribosome concentration is causal for the observed phenotype.

We also agree that epistasis analysis can be informative, but only when the putative downstream factor can be fully removed (Linda S. Huang and Paul W. Sternberg, WormBook 2006; and Avery and Wasserman, PMID: 1365397). Here, RAGA-1-AID represents a partial perturbation of mTORC1 signaling, as full loss of mTORC1 activity (e.g., *daf-15*/raptor mutants) is lethal. The same is true for RPS-26:AID:GFP. Finally, DR represents, by definition, a partial perturbation. Therefore, strictly classical epistasis outcomes are not expected, even if DR was to fully act through mTORC1 signaling.

Instead, in response to this suggestion, we added a new experiment testing the interaction between maternal DR and RAGA-1 depletion across generations. These data show that RAGA-1-AID diminishes the growth difference between DR and AL progeny, consistent with an intergenerational epistatic interaction between maternal diet and mTORC1 signaling. This experiment is now shown in the revised Figure 4E-H and in Supplemental Figure S9.

3) Furthermore does mTOR/RAGA-1 depletion in somatic tissues also influence ribosome content in the oocyte?

We have conducted these measurements and provide this new data in Supplemental Figure S8D-H. We find that the depletion of RAGA-1 by *eft-3p*:Tir1 most strongly reduces RPL-34:mCherry levels in the oocyte immediately distal to the spermatheca, whereas the effect on its levels in the distal germline is weaker. This finding is consistent with our model that maternal treatments can control the ribosome deposition in the embryo and thereby impact progeny phenotypes.

Does it affect the expression of other vitellogenin family proteins in the mother's intestine? Since these proteins are ultimately transferred to the oocyte, they play a critical role in the early development of the progeny.

In our initial submission, we already demonstrated that maternal DR reduces vitellogenin expression in mothers (ln. 100 of the revised manuscript) and their loading into progeny (Supplemental Table S1b). We consider it likely that RAGA-1 depletion similarly influences maternal vitellogenin levels. However, we do not believe these additional measurements are required to support our main conclusions.

We agree that vitellogenins are well-established to affect progeny phenotypes, and we now mention this prior work in more detail in the revised Discussion (e.g., ln. 420). However, the focus of this study is on how maternal diet alters embryonic ribosomal protein concentration and how this change influences early larval growth. By directly reducing ribosomal proteins in embryos using *sun-1p*:Tir1-mediated AID, we show that ribosomal protein depletion is sufficient to slow larval growth, thereby functionally separating ribosome effects from potential vitellogenin-mediated contributions.

Nonetheless, we tested if reduced vitellogenin loading impacts progeny ribosomal proteins by measuring progeny of *rme-2(RNAi)* treated mothers, knocking down the yolk receptor protein. While *rme-2(RNAi)* reduces progeny growth rates, it does not reduce progeny ribosomal protein

levels. These results support the notion that ribosomal proteins are not the only cellular component affecting growth, while demonstrating that ribosomal protein concentrations are not directly determined by vitellogenin abundance.

We provide these data below to the reviewers. For reasons of clarity and focus, we have not included them in the revised manuscript, as they do not directly address our central conclusion, namely that maternal diet intergenerationally regulates progeny ribosomal protein levels, which in turn influences early growth dynamics.

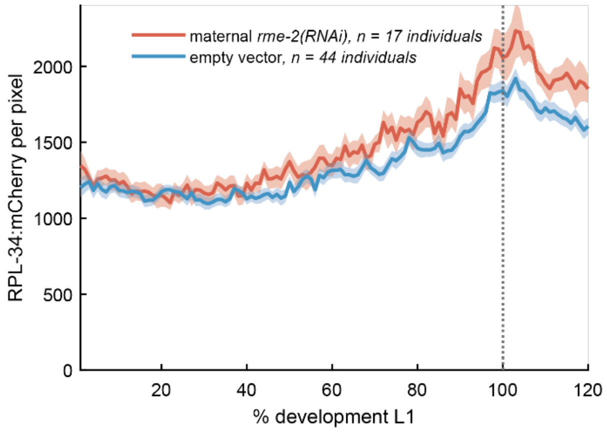

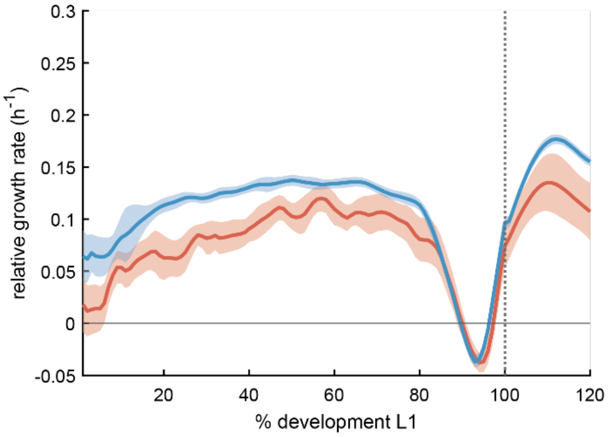

Maternal *rme-2(RNAi)* does not reduce progeny RPL-34:mCherry concentration.

A strain endogenously tagged with RPL-34:mCherry was treated with *rme-2(RNAi)* and progeny were loaded in agarose chambers devoid of RNAi treatment, followed by live imaging to measure growth and RPL-34:mCherry expression. The growth rate of maternally RME-2 depleted animals is strongly reduced, validating efficiency of the RNAi treatment, yet RPL-34:mCherry concentration remained unchanged.

How the RAGA-1 in soma of P0 regulate the expression of ribosome in the F1?

Quantitatively, we previously showed that maternal *eft-3p*:Tir1-mediated RAGA-1 depletion reduces progeny ribosome levels by ~10%, and we now confirm a similar effect with pharynx-specific RAGA-1 depletion.

In the revised manuscript, we expanded the Discussion of potential mechanisms for this soma-to-germline communication, including altered metabolic flux and nutrient provisioning, and canonical signaling pathways (ln. 410ff). Dissecting these mechanisms in detail is beyond the

scope of the current study. Our focus here is to establish that maternal diet and mTORC1 signaling modulate progeny ribosomal protein levels, and that this change has functional consequences for early growth.

4) It is important to analyse the oocyte proteome following mTOR/RAGA-1 depletion in the soma to check the levels of maternally loaded proteins.

We agree that oocyte proteomics would be informative, but current technologies lack the sensitivity required to detect the ~10% changes in ribosomal protein abundance observed in our study. Recent advances in low-input oocyte proteomics (PMID: 38585755) are promising but remain insufficient for the precision needed here. Additionally, our live imaging shows substantial positional variability in ribosome levels across the oocyte queue (Fig. S7D, which would further complicate interpretation of bulk oocyte proteomic measurements.

Importantly, our conclusions are strongly supported by multiple orthogonal measurements (live imaging at hatching, pre-hatching embryonic data, and maternal perturbations), demonstrating that maternal DR alters progeny ribosomal protein levels without requiring direct oocyte proteomics at this stage.

5) The authors should assess the levels of maternally derived proteins under DR and AL conditions in order to substantiate their claim that maternal diet regulates ribosomal protein expression levels in F1, rather than attributing the changes to reduced maternal ribosome loading.

We agree that directly measuring maternally derived ribosomal protein content in early embryos derived from DR vs. AL mothers would be informative. However, isolating intact early embryos from DR mothers at sufficient quality for quantitative imaging is technically challenging: mechanical dissection is inefficient due to the compact uterus under DR, and bleaching compromises the eggshell, making embryos highly sensitive to compression, causing frequent bursting under the cover glass during live imaging.

Instead, we now provide new measurements of embryonic ribosome levels following maternal RAGA-1 depletion. We observe reduced RPL-34:mCherry levels in progeny but no detectable defect in embryonic RPL-34:mCherry synthesis, supporting our model that maternal loading, rather than *de novo* expression in embryos, is altered.

These data further strengthen the conclusion that maternal dietary state modulates the deposition of ribosomes in the progeny.

Minor points

1) Are there other intergenerational phenotypes in response to parental DR? Figure 2D needs to be shown as Figure 1A.

Intergenerational phenotypes of parental DR have been systematically investigated by others. We now cite these works more comprehensively in the introduction of the revised manuscript (references 20-22, ln. 71).

The key advance of our study is the identification of progeny proteome changes, specifically in ribosomes, caused by maternal DR and their effect on progeny growth. We believe this discovery is best presented with the current figure order.

2)	Where is your DR protocol coming from? Is this something you created out of thin air or a published protocol? If published need to cite, if created from nothing need to fully characterize the protocol and its phenotypes. 1A does not have any results but is merely a model. If you are stating that there is a developmental slowing you need to quantify and demonstrate it is statistically significant.

Our protocol is based on published DR methods in liquid culture. The relevant reference has now been added to the revised Methods (line 479).

Additionally, we quantitatively characterize the DR protocol in a new Supplemental Figure S1A. We show that AL-fed animals contain >10 eggs in their uterus already on day 3, whereas the uterus of DR animals is still devoid of eggs on day 4, and holds only few eggs per uterus on day 5, consistent with a reduced adult body size between AL and DR animals.

We retain Figure 1A in the revised manuscript, which depicts the experimental design and sampling schedule.

3)	It would strengthen figure 1 F to show representative images so the readers can see what that fold change actually looks like.

We now show representative images in Supplemental Figure 1H, illustrating the small but highly significant fold change.

4)	I am unclear as to how you use a protein, RPL-34, which is downregulated in response to DR as your marker for Figure 2. Don't you need to use a protein that isn't changing overall level?

We now clarify that RPL-34:mCherry is used for segmentation via a Sobel edge-detection algorithm, which is robust to intensity changes. In a new Supplemental Figure S11, we show that even when images are computationally dimmed by 50%, segmentation accuracy remains very high ($R > 0.99$), demonstrating that RPL-34:mCherry as a marker does not bias our analysis, even if its levels change by 10% between conditions.

Again 2B needs a representative image so the readers can see if an 11% change is actually visible.

We have added representative images, which are now shown in a new Figure 2A.

Might be good to complement with a western blot so that the IF can be a little more quantitative.

While Western blotting can be valuable for confirming large changes in protein abundance, our study focuses on modest quantitative differences that are more precisely resolved using live imaging of fluorescently tagged proteins at single-animal resolution.

Live fluorescence imaging of endogenously tagged proteins provides a linear, internally normalized readout at the level of individual animals and avoids technical variability associated with antibody penetration, epitope accessibility, and batch effects inherent to immunofluorescence (IF) and Western blotting.

The quantitative precision of our method is supported by the narrow inter-individual variance and by statistical analysis across large numbers of animals, as well as by coherence with our orthogonal mass spectrometry-based approach.

5)	I think it would be important to show polysome profiles for your different conditions. Do these look the same or different?

We now provide polysome profiles of strains expressing fluorescently tagged ribosomal proteins in Supplemental Figure 10, confirming that the tagged proteins are incorporated into functional ribosomes. Polysome profiling was primarily used for strain validation, as our study focuses on total ribosome levels rather than translational efficiency.

Assessing ribosome profiles under conditions of maternal dietary restriction could be interesting yet not directly related to the main conclusions of our current manuscript. However, such an experiment would require very large-scale cultures due to low worm density achievable under DR, necessitating new cultivation systems beyond the scope of the current study.

6) All graphs need to start at 0. You can add dashed lines in the axis if you want to focus on a particular region but if it doesn't actually start at 0 you are misleading the reader about the magnitude of these changes.

We appreciate the suggestion and have added dashed lines in the axis where graphs did not start at 0.

7) The mass spectrometry (MS) data shows that members of the vitellogenin (vit) family of proteins are among those most significantly downregulated under DR conditions in mothers. These proteins are maternally loaded into the oocyte and are crucial for early F1 growth and development. Therefore, it is important to investigate whether this effect is influenced by oocyte receptor mutants, such as RME-2(-), which help transport vit proteins into the oocyte. This would confirm that the observed phenotypes in F1 are not a result of lowered maternal vit proteins.

We conducted *rme-2(RNAi)* experiments, which reduced progeny growth rates, but did not change progeny ribosomal protein levels (see Figure provided to reviewers above). Thus, while vitellogenin loading (or other functions of *rme-2*) impacts progeny growth rates, it does not do so through altering ribosome levels.

8) Figure 3 (A-E) clearly shows that depleting maternal rps-26 in the germline (P0) also reduces its level in the progeny (F1), suggesting that the phenotypic changes are due to lower levels of maternally inherited proteins rather than changes in their expression.

We agree with the interpretation that reduced maternal RPS-26 in the germline likely contributes to the inheritance mechanism. Our main finding is that intergenerational variation in core cellular machinery, such as ribosomes, can directly impact progeny phenotypes. This finding complements previous work, which had focused on the role of regulatory molecules, like siRNAs or chromatin modification, in intergenerational inheritance.

9) In Figure 3 (F-H), rpl-22:aid; eft-3p:tir1 was treated with auxin, which degrades rpl-22. However, the authors also checked the level of rpl-34:mCherry. The rationale for this comparison is unclear to me. Why does the level of rpl-34:mCherry decrease following auxin treatment? This data further suggests that somatic ribosomal levels in the parent do not affect progeny development; rather, it is the germline ribosomal proteins that are significant.

We replaced the previous experiment (RPL-22:AID depletion, measuring RPL-34:mCherry) with depletion and quantification of the same ribosomal protein, RPS-26:AID:GFP, to avoid reliance on cross-component interactions. These data are shown in a new Supplemental Figure S3A-E.

We agree that these data are consistent with the interpretation that germline ribosome levels are critical for progeny development and growth. Together with our new data on the effects of

pharynx-specific depletion of mTORC1 signaling, this data aligns with a parsimonious model in which dietary restriction or somatic mTORC1 signaling reduces ribosome production in the germline, thereby lowering maternal ribosome deposition into embryos, and ultimately slowing growth in the progeny.
