# Supplementary material for: Dietary restriction shapes intergenerational ribosome abundance and early growth of Caenorhabditis elegans offspring
Source: PLoS Biol. 2026 Apr 9;24(4):e3003692. doi: 10.1371/journal.pbio.3003692 (PMC13065021; doi:10.1371/journal.pbio.3003692)
Supplement: Attachment — Submitted filename: response to editorial requests.docx [file pbio.3003692.s022.docx]

Dear Dr Towbin,

Thank you for your patience while we considered your revised manuscript "Intergenerational control of ribosomes under dietary restriction" for publication as a Short Report at PLOS Biology. Please accept my sincere apologies for the delays that you have experienced during this round of the peer review process. This revised version of your manuscript has been evaluated by the PLOS Biology editors, the Academic Editor and two of the original reviewers.

Based on the reviews, I am pleased to say that we are likely to accept this manuscript for publication, provided you address the following data and other policy-related requests that I have provided below (A-E):

Thank you for continued interest in our manuscript.

(A) We routinely suggest changes to titles to ensure maximum accessibility for a broad, non-specialist readership. In this case, we would suggest a minor edit to the title, as follows. Please ensure you change both the manuscript file and the online submission system, as they need to match for final acceptance:

“Dietary restriction shapes intergenerational ribosome abundance and early growth of C. elegans offspring”

Thank you. We appreciate and agree to the changed title.

(B) You may be aware of the PLOS Data Policy, which requires that all data be made available without restriction: <https://eur03.safelinks.protection.outlook.com/?url=http%3A%2F%2Fjournals.plos.org%2Fplosbiology%2Fs%2Fdata-availability&data=05%7C02%7Cbenjamin.towbin%40unibe.ch%7C6ba9f649f0af4c4c32d908de68a03371%7Cd400387a212f43eaac7f77aa12d7977e%7C1%7C0%7C639063234417767788%7CUnknown%7CTWFpbGZsb3d8eyJFbXB0eU1hcGkiOnRydWUsIlYiOiIwLjAuMDAwMCIsIlAiOiJXaW4zMiIsIkFOIjoiTWFpbCIsIldUIjoyfQ%3D%3D%7C0%7C%7C%7C&sdata=zFORhseYn3ouKa3QaBbl%2FBdRrm%2F6InsZIntZhfDKeEU%3D&reserved=0>. For more information, please also see this editorial: <https://eur03.safelinks.protection.outlook.com/?url=http%3A%2F%2Fdx.doi.org%2F10.1371%2Fjournal.pbio.1001797&data=05%7C02%7Cbenjamin.towbin%40unibe.ch%7C6ba9f649f0af4c4c32d908de68a03371%7Cd400387a212f43eaac7f77aa12d7977e%7C1%7C0%7C639063234417848530%7CUnknown%7CTWFpbGZsb3d8eyJFbXB0eU1hcGkiOnRydWUsIlYiOiIwLjAuMDAwMCIsIlAiOiJXaW4zMiIsIkFOIjoiTWFpbCIsIldUIjoyfQ%3D%3D%7C0%7C%7C%7C&sdata=XvNkBMw%2BWqb9aQMySZAxq%2B1MJbV3%2Bx4dA78qXsuklI0%3D&reserved=0>

Note that we do not require all raw data. Rather, we ask that all individual quantitative observations that underlie the data summarized in the figures and results of your paper be made available in one of the following forms:

-Supplementary files (e.g., excel). Please ensure that all data files are uploaded as 'Supporting Information' and are invariably referred to (in the manuscript, figure legends, and the Description field when uploading your files) using the following format verbatim: S1 Data, S2 Data, etc. Multiple panels of a single or even several figures can be included as multiple sheets in one excel file that is saved using exactly the following convention: S1_Data.xlsx (using an underscore).

All Data, except for proteomics data, is now provided as Sx Data and cited accordingly in the legend.

-Deposition in a publicly available repository. Please also provide the accession code or a reviewer link so that we may view your data before publication.

Proteomics data has been released for public access on PRIDE (PXD060999)

Regardless of the method selected, please ensure that you provide the individual numerical values that underlie the summary data displayed in the following figure panels as they are essential for readers to assess your analysis and to reproduce it:

Figure 1B-F, 2B-G, 3B-E, 4A-H, S1A, S1C-G, S2, S3A-J, S4A-E, S5A-B, S6A-F, S7A-E, S8A-F, S9A-D, S10A-E, S11A-C

NOTE: the numerical data provided should include all replicates AND the way in which the plotted mean and errors were derived (it should not present only the mean/average values).

Done (see also point C below).

(C) Further to point (B), I note that you have already provided the underlying data in the Source Data file. I have checked and this looks good, but it seems that data for Figures 4E-H and S11A-C are missing from this file?

The missing source data for Figs. 4E-H and Fig S11A-C was added and the data is provided in requested format and cited.

(D) Thank you for providing the mass spectrometry proteomic data in the ProteomeXchange (PXD060999). However, I searched for the deposition and was unable to find it. I would be grateful if you could check whether the accession number is correct or whether the data has been made publicly available at this stage. 

Indeed, the data was previously only available through the provided reviewer login. We have now made the data publicly accessible under the previously indicated identifier number.

(E) Please also ensure that each of the relevant figure legends in your manuscript include information on *WHERE THE UNDERLYING DATA CAN BE FOUND*, and ensure your supplemental data file/s has a legend.

The relevant information has been added to the figure legends.

------------------------------------------------------------------------

As you address these items, please take this last chance to review your reference list to ensure that it is complete and correct. If you have cited papers that have been retracted, please include the rationale for doing so in the manuscript text, or remove these references and replace them with relevant current references. Any changes to the reference list should be mentioned in the cover letter that accompanies your revised manuscript.

In addition to these revisions, you may need to complete some formatting changes, which you will receive in a follow up email. A member of our team will be in touch with a set of requests shortly. If you do not receive a separate email within a few days, please assume that checks have been completed, and no additional changes are required.

We expect to receive your revised manuscript within two weeks.

To submit your revision, please go to <https://eur03.safelinks.protection.outlook.com/?url=https%3A%2F%2Fwww.editorialmanager.com%2Fpbiology%2F&data=05%7C02%7Cbenjamin.towbin%40unibe.ch%7C6ba9f649f0af4c4c32d908de68a03371%7Cd400387a212f43eaac7f77aa12d7977e%7C1%7C0%7C639063234417898915%7CUnknown%7CTWFpbGZsb3d8eyJFbXB0eU1hcGkiOnRydWUsIlYiOiIwLjAuMDAwMCIsIlAiOiJXaW4zMiIsIkFOIjoiTWFpbCIsIldUIjoyfQ%3D%3D%7C0%7C%7C%7C&sdata=y43JeJLgQdoiXlskgHyiTMDBYvrMAJTXF%2BH2V0OQc0s%3D&reserved=0> and log in as an Author. Click the link labelled 'Submissions Needing Revision' to find your submission record. Your revised submission must include the following:
-  a cover letter that should detail your responses to any editorial requests, if applicable, and whether changes have been made to the reference list
-  a Response to Reviewers file that provides a detailed response to the reviewers' comments (if applicable, if not applicable please do not delete your existing 'Response to Reviewers' file.)
-  a track-changes file indicating any changes that you have made to the manuscript.

NOTE: If Supporting Information files are included with your article, note that these are not copyedited and will be published as they are submitted. Please ensure that these files are legible and of high quality (at least 300 dpi) in an easily accessible file format. For this reason, please be aware that any references listed in an SI file will not be indexed. For more information, see our Supporting Information guidelines:
<https://eur03.safelinks.protection.outlook.com/?url=https%3A%2F%2Fjournals.plos.org%2Fplosbiology%2Fs%2Fsupporting-information&data=05%7C02%7Cbenjamin.towbin%40unibe.ch%7C6ba9f649f0af4c4c32d908de68a03371%7Cd400387a212f43eaac7f77aa12d7977e%7C1%7C0%7C639063234417942564%7CUnknown%7CTWFpbGZsb3d8eyJFbXB0eU1hcGkiOnRydWUsIlYiOiIwLjAuMDAwMCIsIlAiOiJXaW4zMiIsIkFOIjoiTWFpbCIsIldUIjoyfQ%3D%3D%7C0%7C%7C%7C&sdata=Os8b7QOsIO08%2FcfwHzItS1kN4aq9rsm6NPpUkTzfJ9U%3D&reserved=0> 

*Published Peer Review History*
Please note that you may have the opportunity to make the peer review history publicly available. The record will include editor decision letters (with reviews) and your responses to reviewer comments. If eligible, we will contact you to opt in or out. Please see here for more details:
<https://eur03.safelinks.protection.outlook.com/?url=https%3A%2F%2Fplos.org%2Fpublished-peer-review-history%2F&data=05%7C02%7Cbenjamin.towbin%40unibe.ch%7C6ba9f649f0af4c4c32d908de68a03371%7Cd400387a212f43eaac7f77aa12d7977e%7C1%7C0%7C639063234417990100%7CUnknown%7CTWFpbGZsb3d8eyJFbXB0eU1hcGkiOnRydWUsIlYiOiIwLjAuMDAwMCIsIlAiOiJXaW4zMiIsIkFOIjoiTWFpbCIsIldUIjoyfQ%3D%3D%7C0%7C%7C%7C&sdata=DzHaJ%2FzdJ%2FInQ1Sm2VgD154%2FvuxvYMGsRKOzQxFa7CI%3D&reserved=0>

*Press*
Should you, your institution's press office or the journal office choose to press release your paper, please ensure you have opted out of Early Article Posting on the submission form. We ask that you notify us as soon as possible if you or your institution is planning to press release the article.

*Protocols deposition*
To enhance the reproducibility of your results, we recommend that if applicable you deposit your laboratory protocols in protocols.io, where a protocol can be assigned its own identifier (DOI) such that it can be cited independently in the future. Additionally, PLOS ONE offers an option for publishing peer-reviewed Lab Protocol articles, which describe protocols hosted on protocols.io. Read more information on sharing protocols at <https://eur03.safelinks.protection.outlook.com/?url=https%3A%2F%2Fplos.org%2Fprotocols%3Futm_medium%3Deditorial-email%26utm_source%3Dauthorletters%26utm_campaign%3Dprotocols&data=05%7C02%7Cbenjamin.towbin%40unibe.ch%7C6ba9f649f0af4c4c32d908de68a03371%7Cd400387a212f43eaac7f77aa12d7977e%7C1%7C0%7C639063234418035283%7CUnknown%7CTWFpbGZsb3d8eyJFbXB0eU1hcGkiOnRydWUsIlYiOiIwLjAuMDAwMCIsIlAiOiJXaW4zMiIsIkFOIjoiTWFpbCIsIldUIjoyfQ%3D%3D%7C0%7C%7C%7C&sdata=%2BXETrSswg2kL7%2BubK0LFrAIUojy5Efb0TIPePwoQEGY%3D&reserved=0>


Please do not hesitate to contact me should you have any questions.

Best regards,

Richard

Richard Hodge, PhD
Senior Editor, PLOS Biology
rhodge@plos.org
